# Heterogeneity of resting-state EEG features in juvenile myoclonic epilepsy and controls

**DOI:** 10.1093/braincomms/fcac180

**Published:** 2022-07-08

**Authors:** Amy Shakeshaft, Petroula Laiou, Eugenio Abela, Ioannis Stavropoulos, Mark P Richardson, Deb K Pal, BIOJUME Consortium, Alessandro Orsini, Alice Howell, Alison Hyde, Alison McQueen, Almu Duran, Alok Gaurav, Amber Collingwood, Amy Kitching, Amy Shakeshaft, Anastasia Papathanasiou, Andrea Clough, Andrew Gribbin, Andrew Swain, Ann Needle, Anna Hall, Anna Smith, Anne Macleod, Asyah Chhibda, Beata Fonferko-Shadrach, Bintou Camara, Boyanka Petrova, Carmel Stuart, Caroline Hamilton, Caroline Peacey, Carolyn Campbell, Catherine Cotter, Catherine Edwards, Catie Picton, Charlotte Busby, Charlotte Quamina, Charlotte Waite, Charlotte West, Ching Ching Ng, Christina Giavasi, Claire Backhouse, Claire Holliday, Claire Mewies, Coleen Thow, Dawn Egginton, Debbie Dickerson, Debbie Rice, Dee Mullan, Deirdre Daly, Dympna Mcaleer, Elena Gardella, Elma Stephen, Eve Irvine, Eve Sacre, Fan Lin, Gail Castle, Graham Mackay, Halima Salim, Hannah Cock, Heather Collier, Helen Cockerill, Helen Navarra, Hilda Mhandu, Holly Crudgington, Imogen Hayes, Ioannis Stavropoulos, Jacqueline Daglish, Jacqueline Smith, Jacqui Bartholomew, Janet Cotta, Javier Peña Ceballos, Jaya Natarajan, Jennifer Crooks, Jennifer Quirk, Jeremy Bland, Jo Sidebottom, Joanna Gesche, Joanne Glenton, Joanne Henry, John Davis, Julie Ball, Kaja K Selmer, Karen Rhodes, Kelly Holroyd, Kheng Seang Lim, Kirsty O’Brien, Laura Thrasyvoulou, Linetty Makawa, Lisa Charles, Lisa Richardson, Liz Nelson, Lorna Walding, Louise Woodhead, Loveth Ehiorobo, Lynn Hawkins, Lynsey Adams, Margaret Connon, Marie Home, Mark Baker, Mark Mencias, Mark P Richardson, Mark Sargent, Marte Syvertsen, Matthew Milner, Mayeth Recto, Michael Chang, Michael O'Donoghue, Michael Young, Munni Ray, Naim Panjwani, Naveed Ghaus, Nikil Sudarsan, Nooria Said, Owen Pickrell, Patrick Easton, Paul Frattaroli, Paul McAlinden, Rachel Harrison, Rachel Swingler, Rachel Wane, Rebecca Ramsay, Rikke S Møller, Robert McDowall, Rosie Clegg, Sal Uka, Sam White, Samantha Truscott, Sarah Francis, Sarah Tittensor, Sarah-Jane Sharman, Seo-Kyung Chung, Shakeelah Patel, Shan Ellawela, Shanaz Begum, Sharon Kempson, Sonia Raj, Sophie Bayley, Stephen Warriner, Susan Kilroy, Susan MacFarlane, Thomas Brown, Tinashe Samakomva, Tonicha Nortcliffe, Verity Calder, Vicky Collins, Vicky Parker, Vivien Richmond, William Stern, Zena Haslam, Zuzana Šobíšková, Amit Agrawal, Amy Whiting, Andrea Pratico, Archana Desurkar, Arun Saraswatula, Bridget MacDonald, Choong Yi Fong, Christoph P Beier, Danielle Andrade, Darwin Pauldhas, David A Greenberg, David Deekollu, Deb K Pal, Dina Jayachandran, Dora Lozsadi, Elizabeth Galizia, Fraser Scott, Guido Rubboli, Heather Angus-Leppan, Inga Talvik, Inyan Takon, Jana Zarubova, Jeanette Koht, Julia Aram, Karen Lanyon, Kate Irwin, Khalid Hamandi, Lap Yeung, Lisa J Strug, Mark Rees, Markus Reuber, Martin Kirkpatrick, Matthew Taylor, Melissa Maguire, Michalis Koutroumanidis, Muhammad Khan, Nick Moran, Pasquale Striano, Pronab Bala, Rahul Bharat, Rajesh Pandey, Rajiv Mohanraj, Rhys Thomas, Rosemary Belderbos, Seán J Slaght, Shane Delamont, Shashikiran Sastry, Shyam Mariguddi, Siva Kumar, Sumant Kumar, Tahir Majeed, Uma Jegathasan, William Whitehouse

**Affiliations:** Department of Basic & Clinical Neuroscience, Institute of Psychiatry, Psychology & Neuroscience, King’s College London, London, UK; MRC Centre for Neurodevelopmental Disorders, King’s College London, London, UK; Department of Biostatistics and Health Informatics, Institute of Psychiatry, Psychology & Neuroscience, King’s College London, London, UK; Department of Basic & Clinical Neuroscience, Institute of Psychiatry, Psychology & Neuroscience, King’s College London, London, UK; King’s College Hospital, London, UK; Department of Basic & Clinical Neuroscience, Institute of Psychiatry, Psychology & Neuroscience, King’s College London, London, UK; MRC Centre for Neurodevelopmental Disorders, King’s College London, London, UK; King’s College Hospital, London, UK; Department of Basic & Clinical Neuroscience, Institute of Psychiatry, Psychology & Neuroscience, King’s College London, London, UK; MRC Centre for Neurodevelopmental Disorders, King’s College London, London, UK; King’s College Hospital, London, UK; Evelina London Children’s Hospital, London, UK; Department of Basic & Clinical Neuroscience, Institute of Psychiatry, Psychology & Neuroscience, King’s College London, London, UK

**Keywords:** EEG, epilepsy, networks, heterogeneity, biomarkers

## Abstract

Abnormal EEG features are a hallmark of epilepsy, and abnormal frequency and network features are apparent in EEGs from people with idiopathic generalized epilepsy in both ictal and interictal states. Here, we characterize differences in the resting-state EEG of individuals with juvenile myoclonic epilepsy and assess factors influencing the heterogeneity of EEG features. We collected EEG data from 147 participants with juvenile myoclonic epilepsy through the Biology of Juvenile Myoclonic Epilepsy study. Ninety-five control EEGs were acquired from two independent studies [Chowdhury *et al*. (2014) and EU-AIMS Longitudinal European Autism Project]. We extracted frequency and functional network-based features from 10 to 20 s epochs of resting-state EEG, including relative power spectral density, peak alpha frequency, network topology measures and brain network ictogenicity: a computational measure of the propensity of networks to generate seizure dynamics. We tested for differences between epilepsy and control EEGs using univariate, multivariable and receiver operating curve analysis. In addition, we explored the heterogeneity of EEG features within and between cohorts by testing for associations with potentially influential factors such as age, sex, epoch length and time, as well as testing for associations with clinical phenotypes including anti-seizure medication, and seizure characteristics in the epilepsy cohort. *P*-values were corrected for multiple comparisons. Univariate analysis showed significant differences in power spectral density in delta (2–5 Hz) (*P* = 0.0007, hedges’ g = 0.55) and low-alpha (6–9 Hz) (*P* = 2.9 × 10^−8^, g = 0.80) frequency bands, peak alpha frequency (*P* = 0.000007, g = 0.66), functional network mean degree (*P* = 0.0006, g = 0.48) and brain network ictogenicity (*P* = 0.00006, g = 0.56) between epilepsy and controls. Since age (*P* = 0.009) and epoch length (*P* = 1.7 × 10^−8^) differed between the two groups and were potential confounders, we controlled for these covariates in multivariable analysis where disparities in EEG features between epilepsy and controls remained. Receiver operating curve analysis showed low-alpha power spectral density was optimal at distinguishing epilepsy from controls, with an area under the curve of 0.72. Lower average normalized clustering coefficient and shorter average normalized path length were associated with poorer seizure control in epilepsy patients. To conclude, individuals with juvenile myoclonic epilepsy have increased power of neural oscillatory activity at low-alpha frequencies, and increased brain network ictogenicity compared with controls, supporting evidence from studies in other epilepsies with considerable external validity. In addition, the impact of confounders on different frequency-based and network-based EEG features observed in this study highlights the need for careful consideration and control of these factors in future EEG research in idiopathic generalized epilepsy particularly for their use as biomarkers.

## Teaser

Shakeshaft *et al*. report differences in the resting-state EEG of patients with Juvenile Myoclonic Epilepsy compared with healthy controls, particularly in features relating to alpha activity. They also identify factors influencing these EEG features, such as age, time of day and specific seizure characteristics.

## Graphical abstract

**Graphical abstract:**
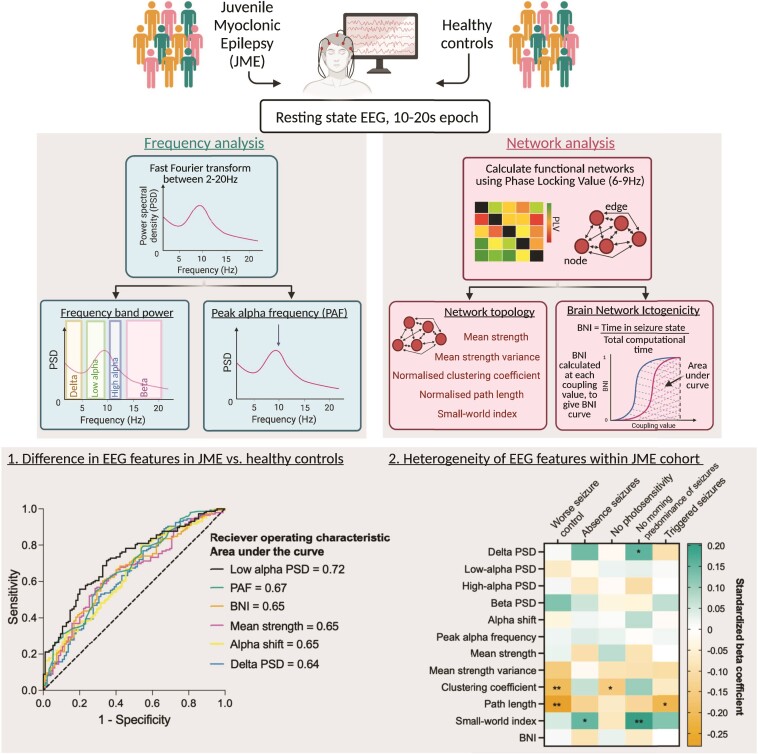

## Introduction

An abnormal ictal EEG is the hallmark of epilepsy and the presence of epileptiform discharges, such as spontaneous or provoked 3–6 Hz spike and waves, are routinely used to identify idiopathic generalized epilepsy (IGE), including juvenile myoclonic epilepsy (JME), in the clinical diagnostic process. A normal background EEG is required for a JME diagnosis,^1^ with no generalized or focal slowing.^2^ However, recent advances in computational analysis of EEG have shown that the interictal EEG of patients with IGE show differences compared with healthy controls.

Several studies on the EEG power spectrum in individuals with epilepsy have focused on alterations in the alpha rhythm: the oscillatory activity most apparent at posterior EEG electrodes at around 8–13 Hz. Early EEG studies reported shifts in alpha power from a higher frequency (around 8–13 Hz) to a lower frequency (6–8 Hz) in patients with epilepsy,^3,4^ with recent studies confirming these findings using robust and standardized quantitative methodology in patients with focal and generalized epilepsy, and controlling for anti-seizure medication (ASM).^5–7^ Furthermore, there is evidence for a decreased peak alpha frequency in patients with epilepsy^8^ and their asymptomatic first-degree relatives.^9^ The alpha rhythm is of particular interest in IGE due to evidence of its basis in cortico-thalamic interactions,^10^ which are central to the generation of generalized seizures/spike-wave discharges^11,12^ and functionally and structurally altered in JME.^13,14^ There is also evidence of abnormalities in other EEG frequency bands in IGE, such as increased theta^15,16^ and beta power.^16^

Furthermore, functional networks derived from EEG or MEG (magnetoencephalography) activity have altered topology in IGE, as quantified using graph theory,^17–21^ with evidence for the most extensive changes in functional connectivity existing in JME compared with other IGE syndromes.^22^ Whilst the reported differences in network topology are variable, increased clustering coefficient (indicating increased regularity of networks) is a somewhat consistent finding across studies.^20^ The variability of results in studies of functional connectivity in IGE likely comes from factors such as heterogeneity of patient groups, ages of participants, frequency bands in which functional networks are derived, epoch length and the use of different network types (binary/weighted and undirected/directed), as well as methods used for their computation. Furthermore, there is little known about the external validity and reproducibility of these network topology measures across different cohorts at different sites.

In addition to these static models of functional brain networks, dynamic models can aid the understanding of seizure mechanisms by modelling transitions of brain networks from stable, interictal states to ictal states, and the parameters that are the most influential in this transition. Several dynamic models have been proposed as biomarkers of epilepsy, including the integration of global network structure and local node coupling into a phase oscillator model, which showed 57% sensitivity (given 100% specificity) and 66% specificity (given 100% sensitivity) as a biomarker of IGE.^19^ Brain network ictogenicity (BNI) is a computational method that quantifies the ability of a network to generate seizures. It models the dynamics at each node of a functional network using a dynamical model^23,24^ and measures the time each network spends in a seizure-like state. Therefore, BNI depicts the propensity of a given functional network to generate seizure activity. This method has been used to predict surgical outcomes^24,25^ and aid with resection site choice in patients with focal epilepsy,^26^ as well as differentiating between focal and generalized epilepsies^18^ when applied to EEG data. Furthermore, using MEG data, Lopes *et al*.^27^ showed that BNI can act as a biomarker of JME with 73% classification accuracy. However, this model has not yet been explored in EEG data of patients with JME.

There have been variable findings as to whether these EEG features differ within IGE cohorts, according to specific phenotypes. Abela *et al*.^5^ showed that patients with poor seizure control (both IGE and focal epilepsy) had an increased shift of alpha oscillatory activity from high frequencies to low frequencies compared to those with good seizure control and healthy controls. However, similar work by Pegg *et al*.^7^ found no difference in spectral power between patients with well-controlled IGE and drug-resistant IGE. In addition, there were no differences in functional network topology in the same IGE cohort.^21^ However, studies in patients with focal epilepsy have shown that dynamic network measures, such as BNI, show promise as a predictor of prognostic outcome,^24,25^ as well as indicating differences in seizure/epilepsy type.^18^

The aim of this study is to assess and compare a range of resting-state interictal EEG features in a large cohort of individuals with JME to healthy controls, using EEGs collected across multiple sites. Further we will investigate the reliability and heterogeneity of these measures in these cohorts and any clinical factors influencing them. Table 1 shows the hypothesized direction of change of each EEG measure in JME compared with controls based on evidence from previous literature.

**Table 1:** Hypothesized change of direction of EEG features in JME compared with controls

EEG measure	Hypothesized direction of change in JME compared with controls	Previous evidence
**Delta PSD**	—	—
**Low-alpha PSD**	↑	Abela *et al.* (2019)^5^ Pegg *et al.* (2020)^7^
**High-alpha PSD**	↓	Abela *et al.* (2019)^5^
**Beta PSD**	↑	Glaba *et al.* (2020)^16^
**Peak alpha frequency**	↓	Larsson & Kostov (2005)^8^ Yaakub et al. (2020)^9^
**Log_10_ alpha shift**	↑	Abela *et al.* (2019)^5^
**Mean strength**	↑	Chowdhury *et al.* (2014)^17^
**Mean strength variance**	↑	Chowdhury *et al.* (2014)^17^
**Clustering coefficient**	↑	Chowdhury *et al.* (2014)^17^
**Path length**	—	—
**Small-world index**	↓	Lee *et al.* (2020)^22^
**BNI**	↑	Lopes *et al.* (2021)^27^

BNI = brain network ictogenicity; JME = juvenile myoclonic epilepsy; PSD = power spectral density.

## Methods

### Participants

#### Biology of juvenile myoclonic epilepsy study

The Biology of Juvenile Myoclonic Epilepsy (BIOJUME) Consortium is an international cross-sectional study, spanning 72 sites from 12 countries focused on young people and adults with JME.^28^ BIOJUME collects clinical and EEG data from patients with JME.

#### Participant recruitment

Inclusion criteria for this study are based on Avignon Class II consensus criteria for the diagnosis of JME:^1^ (i) age of myoclonus onset 6–25 years; (ii) seizures comprising predominant or exclusive early morning myoclonus of upper extremities and (iii) EEG interictal generalized spikes/polyspike and waves with normal background. Participants aged between 6 and 55 years were included. Exclusion criteria were as follows: (i) myoclonus only associated with carbamazepine or lamotrigine therapy; (ii) EEG showing predominant focal interictal epileptiform discharges or abnormal background; (iii) any evidence of progressive or symptomatic myoclonus epilepsy or focal seizures; (iv) global learning disability; (v) dysmorphic features or (vi) unable to provide informed consent.

### Clinical data collection

Clinical data were collected by study site researchers face-to-face and augmented by clinical records and EEG reports. The data set included general demographics and health information, epilepsy history, including seizure types, seizure frequency, drug/lifestyle interventions and the presence of a photoparoxysmal response (PPR).^29^ Sites uploaded clinical data onto a secure central REDCap (Research Electronic Data Capture) database.^30,31^

#### Phenotyping

A phenotyping panel, comprising seven epilepsy experts, then evaluated the diagnosis of JME according to the inclusion criteria.

#### Seizure prognosis

To test associations of EEG features with seizure prognosis, we categorized participants based on their answers to two questions (using the internationally agreed definitions of seizure freedom and drug resistance as a basis^32^): (i) whether they had been free from seizures over the past year and (ii) current ASM therapy, categorized as either no drug therapy, monotherapy (not necessarily the first appropriate ASM), dual therapy, or drug-resistant (two or more ASM failures). Based on answers to these two questions, participants were categorized based on their seizure control:

- **Good prognosis**—those who are seizure-free on monotherapy or no drug therapy,
- **Moderate prognosis**—those who are either seizure-free on ≥2 ASMs or not seizure-free on 0/1 ASM, or
- **Poor prognosis**—those who are not seizure-free on ≥2 ASMs (drug-resistant).

Using this classification, eight individuals were unable to be categorized, due to missing data.

### EEG data collection

#### Juvenile myoclonic epilepsy

Routine clinical EEGs were collected for participants from each BIOJUME study site. Natus Xltek or Nicolet systems were used for clinical EEG data collection. Sites used between 19 and 25 scalp electrodes placed according to the 10–20 system, apart from Danish EEGs which used the modified 10–20 system whereby electrodes T3/T4 are replaced by T7/T8 and electrodes T5/T6 are replaced by P7/P8. EEGs were sent securely to a central site where one (or two where possible) 10–20 s resting-state, eyes-closed, awake segments with clear background oscillatory activity were selected by a trained EEG neurophysiologist. Epoch lengths between 10 and 20 s was chosen based on evidence of stability of EEG network measures in epochs over 10–12 s,^33^ and since previous analysis of EEG measures in IGE found differences to controls using 20 s epochs.^5,17,19,34^ A single, fixed length was not used due to our aim to investigate the effect of epoch length on EEG features, and also the difficulties in finding 20 s epochs with clear background alpha activity with few artefacts in clinical EEGs, as noted in Chowdhury *et al.*^17^

#### Control

Control EEG data were acquired from Chowdhury *et al*.^17^ study and the EU-AIMS Longitudinal European Autism Project (LEAP) study.^35^ Controls were not age-matched to the JME cohort to maximize the available sample, but any differences in demographics were adjusted for in analysis.

##### Chowdhury controls

Healthy controls with no personal or family history of neurological or psychiatric diseases were recruited via a local research participant database. Participants were excluded if they had any other neuropsychiatric condition or a full-scale IQ <70. Ethical approval was obtained from King’s College Hospital Research Ethics Committee (08/H0808/157). Written informed consent was obtained from all participants. EEGs were acquired as previously described in Chowdhury *et al.*^17^ Briefly, conventional 19 channel 10–20 layout scalp EEG were collected (sampling rate 256 Hz, filtered band-pass 0.3–70 Hz) using a NicoletOne system. A single 20 s epoch was selected which included continuous dominant background rhythm with eyes-closed, without any artefacts or patterns indicating drowsiness or arousal.

##### Longitudinal European Autism Project controls

Another control group was recruited as part of the EU-AIMS LEAP.^35^ Only healthy control EEG data were used. Participants were typically developing individuals aged 6–30 years. Two minutes of eyes-closed resting-state EEG were recorded per participant. Data used for this study was acquired from three sites: King’s College London (KCL, United Kingdom) (*N* = 2), University Campus BioMedico (UCBM, Rome, Italy) (*N* = 15) and University Medical Centre Utrecht (UMCU, Netherlands) (*N* = 40). The following EEG systems were employed: Brainvision (KCL), Biosemi (UMCU) and Micromed (UCBM), with sampling frequencies of 5000 Hz (KCL), 2048 Hz (UMCU) and 256–1000 Hz (UCBM). All sites used 10–20 layout caps, with 60–64 electrodes. Eyes-closed, resting-state segments were marked on the EEGs and from these segments, 10–20 s resting-state, eyes-closed, awake segments with clear background oscillatory activity were selected.

### EEG pre-processing

**Figure 1:**
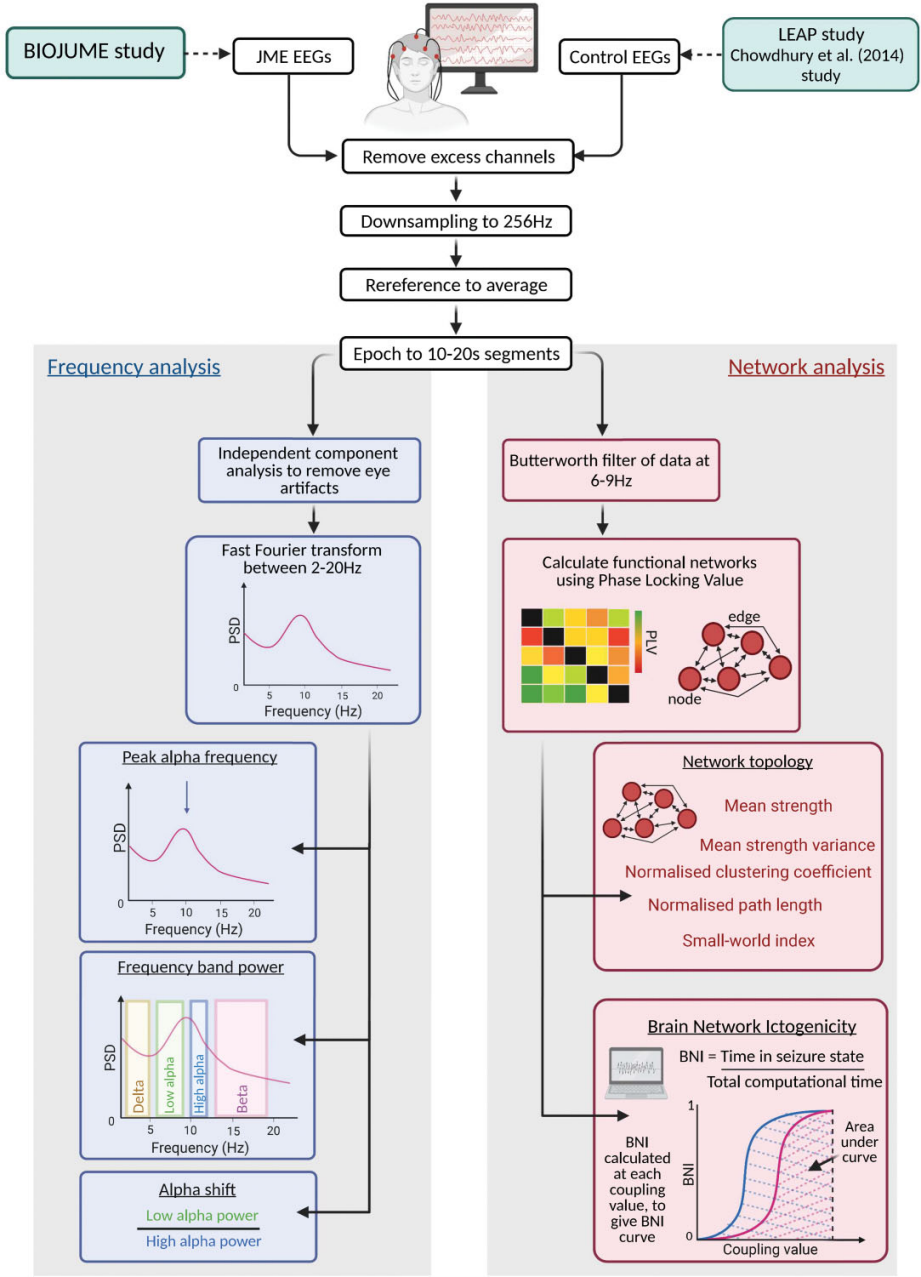
**Summary of methods for EEG processing and analysis.** BIOJUME = Biology of Juvenile Myoclonic Epilepsy; BNI = brain network ictogenicity; JME = juvenile myoclonic epilepsy; LEAP = Longitudinal European Autism Project

An overview of the EEG processing and analysis pipeline can be seen in Fig. 1.

We carried out pre-processing using Fieldtrip^36^ or custom written MATLAB (Version R2019a)^37^ scripts. Analysis was only undertaken on the following 19 EEG channels: Fp1, Fp2, Fz, F3, F7, F4, F8, T3, C3, Cz, C4, T4, T5, P3, Pz, P4, T6, O1, O2 (channels labelled T7, T8, P7, P8 were used for Danish EEGs as are in the same location as T3, T4, T5, T6 in the traditional 10–20 system). Any additional channels were removed. Data were re-referenced to average montage and resampled to the minimum sampling rate of all data (256 Hz). EEGs were then segmented at the pre-defined epochs (10–20 s eyes-closed, resting-state sections). Epochs across all three subgroups [(i) BIOJUME (JME); (ii) LEAP controls and (iii) Chowdhury controls] were independently reviewed and confirmed to ensure consistency in epoch choice between subgroups.

### EEG features

Twelve measures (six frequency-based and six network-based) were extracted from JME and control EEGs. Full methodology for the calculation of EEG features is presented in Supplementary Methods but outlined briefly below.

#### Frequency-based

Independent component analysis (ICA) was used to remove any remaining artefacts existing in the EEG between 2 and 20 Hz and re-referenced to average montage. We calculated the mean relative power spectral density (PSD) in (i) delta (2–5 Hz), (ii) low-alpha (6–9 Hz), (iii) high-alpha (10–11 Hz), (iv) beta (12–19 Hz) frequency bands, (v) the peak alpha frequency (PAF) and (vi) the shift in alpha PSD from high to low-alpha (alpha shift).

#### Network-based

Functional networks were inferred using phase-locking value (PLV) on EEG epochs Butterworth band-pass filtered between 6 and 9 Hz. Five measures were used to characterize functional network topology: (i) mean strength, (ii) mean strength variance, (iii) average clustering coefficient, (iv) average characteristic path length and (v) small-world index. In addition, the propensity of networks to generate seizure-like dynamics was modelled using the framework of BNI.^24,25^ Larger BNI values indicate that the brain network has a higher tendency to transmit from normal to seizure-like dynamics.

### Statistical analysis procedure

Statistical analysis was carried out in SPSS^38^ software and graphics produced in GraphPad Prism. Prior to statistical testing, violations of test assumptions were checked, and statistical tests chosen accordingly. For all multiple linear regression models, variance inflation factors (VIFs) were checked to ensure no problematic multicollinearity (VIF < 10). Transformations and removal of outliers were done where required. Hedge’s g was used to calculate effect sizes.

#### Demographics, clinical characteristics and EEG details

Differences in age, epoch length and EEG time between cohorts were testing using two-tailed Mann–Whitney tests. Differences in sex distribution were tested using two-tailed χ^2^ tests.

#### EEG features in juvenile myoclonic epilepsy compared with controls

Disparities in EEG features in JME and controls were first tested univariately using Mann–Whitney tests. *P*-values were corrected for multiple comparisons for the four frequency bands in PSD analysis and for the five measures of network topology using Bonferroni–holm correction. Kruskal–Wallis tests were then used to test for differences in EEG features between the two control groups (LEAP & Chowdhury controls) and JME, again corrected for multiple comparisons for the four frequency bands and for the five measures of network topology using Bonferroni–holm correction. If these tests were significant (corrected *P*-value <0.05), Bonferroni–holm corrected Mann–Whitney tests were carried out between each of the three groups.

We then performed multiple linear regression analysis of each EEG feature to investigate differences between JME and control groups whilst accounting for the appropriate covariates (sex, age, epoch length, EEG time, ASM treatment, control subgroup). For EEG features where there was an opposing direction of effect between the two control groups and JME, LEAP controls and Chowdhury controls were added separately into the regression models as dummy variables (JME = 0/LEAP = 1; JME = 0/Chowdhury = 1). This enabled differences between each control subgroup to be detected rather than combining control subgroups. Similarly, for EEG measures which differed between JME patients not on ASM therapy (untreated) and those on ASM therapy (treated), these were also added separately to the model as dummy variables (control = 0/untreated JME = 1; control = 0/treated JME = 1). Estimated marginal means of EEG measures for subgroups were obtained from these multivariable regression models.

#### Receiver operating characteristic analysis

We calculated receiver operating characteristic (ROC) curves for EEG features which were significantly different in univariate JME versus control analysis, to quantify their ability to discriminate JME and control EEGs. An optimal cut-off point was obtained for the EEG feature with the highest ROC area under the curve (AUC), using the value with the highest sensitivity and specificity values. Positive and negative predictive values were calculated using standard methods.^39^

#### Heterogeneity of EEG features within and between cohorts and recording conditions

##### Influence of confounding factors on EEG features

Associations between potential confounders and EEG features were tested using Spearman’s rank tests for continuous variables (age, EEG time, epoch length) and two-tailed Mann–Whitney tests (stratified by JME and control groups) for sex. For analysis of EEG time, 11 JME EEGs with recording times between 23.35 and 00.21 were excluded, leaving only EEGs with recording times between 8:00–20:00. Differences in EEG features between JME individuals not on ASM therapy (untreated) and those on ASM therapy (treated) at the time of the EEG were tested using multiple linear regression models of each EEG feature including age as a covariate. A one-way analysis of covariance (ANCOVA) was used to test for differences in EEG features between BIOJUME recruitment countries (*n* = 7), controlling for age and epoch length.

##### Associations with clinical phenotypes in juvenile myoclonic epilepsy

Multiple linear regression analysis was performed to test for associations of EEG features with clinical variables (seizure type, seizure control, PPR) within the JME cohort. Estimated marginal means of EEG measures for JME subgroups were obtained from these multivariable regression models.

Since sodium valproate is the most effective ASM in JME, we also performed additional analysis of the association between seizure control and EEG features in the subset of patients who report having used sodium valproate to control their seizures to check if a lack of sodium valproate use in females was creating additional heterogeneity in the drug-resistant subgroup (results presented in Supplementary material).

##### Test–retest reliability of EEG measures

Two methods were used to investigate the test–retest reliability of each EEG measure: (i) Spearman’s rank correlation coefficient and (ii) intra-class correlation (ICC). The test–retest reliability of EEG features were tested first between epochs within the same EEG recording session (*between-epoch*) and second, between EEGs from different recording sessions but in the same ASM treatment state (both EEG taken whilst untreated or both while treated) (*between-EEG*). For the between-epoch analysis a two-way mixed model ICC with absolute agreement was used, using single measures. For the between-EEG analyses, a two-way mixed model ICC with absolute agreement, using average measures was used. To control for the effect of epoch length, we ran further analysis comparing EEG measures between epochs of the same/similar ( ± 5 s) length.

#### Ethical approval

BIOJUME is funded by the Canadian Institutes of Health Research (MOP-142405) and received ethical approval from the National Health Service (NHS) Health Research Authority (South Central—Oxford C Research Ethics Committee, reference 16/SC/0266) and the Research Ethics Board of the Hospital for Sick Children, Toronto (REB#1000033784). Local ethical approvals were also held for all international sites. All procedures complied with appropriate regulatory requirements and ethical principles in line with the Declaration of Helsinki. Informed consent was obtained and documented for all participants. Assent was obtained from minors (under 16), and informed consent was obtained on their behalf by a parent or legally appropriate guardian. All data from participants were de-identified before entry onto the central database.

#### Data availability

The data supporting the findings of this study are available from the corresponding author upon reasonable request.

## Results

### Demographics, clinical characteristics and EEG details

One hundred and ninety-four EEGs from 147 individuals with JME and 95 EEGs from 95 control participants were included in this study. Demographics from both groups and EEG details are presented in Table 2, with the JME group further stratified by ASM treatment status and the control group broken down by data origin. There were significant age (U = 5156, *P* = 0.003) and sex (χ^2^ = 7.3, *P* = 0.007) differences between JME and control cohorts. Furthermore, EEG time was significantly different between JME and controls, with JME EEGs occurring earlier than control EEGs (U = 661, *P* = 2.6 × 10^−13^). Whilst all EEG epochs were between 10 and 20 s long, control epochs were on average significantly longer than JME epochs (U = 4353, *P* = 1.7 × 10^−8^). The clinical characteristics of the JME cohort are presented in Table 3.

**Table 2:** Demographics of JME and control participants included in the EEG study

	JME			Control			JME total versus control total		JME untreated versus treated		LEAP versus Chowdhury	
	Total	Untreated	Treated	Total	LEAP	Chowdhury	Test statistic	*P*-value	Test statistic	*P*-value	Test statistic	*P*-value
***N* EEGs**	194	72	99	95	57	38	—	—	—	—	—	—
***N* individuals**	147	60	72	95	57	38	—	—	—	—	—	—
***N* female (%)**	83 (60%)	36 (61%)	42 (62%)	40 (42%)	20 (35%)	20 (53%)	χ^2^(1) = 7.3	**0.007**	χ^2^(1) = 0.007	0.93	χ^2^(1) = 2.9	0.09
**Mean age at EEG** ± **SD**^a^	20.5 ± 7.7	16.8 ± 5.2	23.7 ± 8.3	23.4 ± 9.5	18.8 ± 6.6	30.3 ± 8.9	U = 5156	**0.003**	U = 953	**8.0 × 10^−8^**	U = 297	**2.2 × 10^−9^**
**Mean epoch length** ± **SD (s)**^a^	16.2 ± 4.3	15.9 ± 4.4	16.5 ± 4.2	19.2 ± 1.9	18.8 ± 2.3	20.0 ± 0.0	U = 4355	**1.7 × 10^−8^**	U = 1965	0.34	U = 722	**0.00009**
**Median EEG time** ± **interquartile range**^a^	10:44 ± 3:08	10:54 ± 3:12	10:35 ± 2:23	14:52 ± 4:14	14:52 ± 4:14	—	U = 661	**2.6 × 10^−13^**	U = 1661	0.27	—	—

a Duplicate EEGs (multiple for one participant) were excluded from descriptive statistics. The most recent EEG was chosen if both were in the same treatment condition, if in different treatment conditions, untreated EEGs were used. *P*-values for continuous dependent variables are from Mann–Whitney U tests and for categorical variables from χ^2^ tests (bold text indicates *P* < 0.05). Test statistics and *P*-values for JME total versus control total, JME subgroups untreated versus treated and control subgroups LEAP versus Chowdhury are presented on the right of the table. JME = juvenile myoclonic epilepsy.

**Table 3:** Demographics and clinical characteristics of participants included in this study. Percentage denominators are adjusted for missing data

Demographic/clinical characteristic	Summary statistic
**Total JME cohort** (*N*, %)	147 (100%)
**Females** (*N*, %)	83 (60%)
**Age at EEG** (years, mean ± SD)	20.5 ± 7.7
**Age at clinical data collection** (years, mean ± SD)	24.8 ± 7.9
**Years between-EEG and clinical data collection** (median, range)	3.7 (−1.4–12.3)
**Seizure control**	
Good (*N*, %)	56 (40%)
Moderate (*N*, %)	40 (29%)
Poor (*N*, %)	43 (31%)
**Seizure types**	
Generalized tonic–clonic seizures (GTCS) (*N*, %)	127 (91%)
Absence seizures (*N*, %)	60 (44%)
**Morning predominance of seizures** (*N*, %)	100 (76%)
**Age at myoclonus seizure onset** (years, mean ± SD)	14.5 ± 3.4
**Seizure duration (at the time of EEG)** (years, mean ± SD)	6.6 ± 7.8
**Self-reported triggered seizures** (*N*, %)	97 (72%)
**Photoparoxysmal response** (*N*, %)	52 (43%)

JME = juvenile myoclonic epilepsy.

### Differences in EEG features between juvenile myoclonic epilepsy and controls

Table 4 shows the results of univariate tests of EEG features between controls and JME. Average relative PSD plots for both JME and controls are displayed in Supplementary Fig. 3. Relative delta PSD and PAF are significantly lower in JME than controls whilst relative low-alpha PSD, alpha shift, mean strength and BNI are significantly higher in JME in univariate analysis. Relative low-alpha PSD has the greatest effect size of g = 0.72. Analysis with stratified control subgroups (Table 4) shows an opposite direction of effect in LEAP and Chowdhury control subgroups compared with JME for relative beta PSD, mean strength variance, average clustering coefficient, average characteristic path length and small-world index.

**Table 4:** Summary of *P*-values from univariate statistical tests on EEG features between groups

EEG feature	Control versus JME				Stratified control groups			
	Uncorrected *P*-values	Bonferroni–holm corrected *P*-values	Hedge’s g	ROC AUC	Comparison	Mean diff	Uncorrected *P*-values	Bonferroni–holm corrected *P*-values
Relative Delta PSD	**0**.**00024**	**0**.**00072**	0.55	0.64	Difference between groups (Kruskal–Wallis)	**—**	**0**.**0011**	**0**.**0023**
					LEAP v. JME (Mann–Whitney)	0.0014	**0**.**0024**	**0**.**0072**
					Chowdhury versus JME (Mann–Whitney)	0.0020	**0**.**0064**	**0**.**013**
					LEAP versus Chowdhury (Mann–Whitney)	−0.00063	0.64	0.64
Relative Low-alpha PSD	**7.2 × 10^−9^**	**2.9 × 10^−8^**	0.80	0.72	Difference between groups (Kruskal–Wallis)	**—**	**6.7 × 10^−9^**	**2.7 × 10^−8^**
					LEAP versus JME (Mann–Whitney)	−0.0023	**0**.**00008**	**0**.**00016**
					Chowdhury versus JME (Mann–Whitney)	−0.0035	**7.2 × 10^−8^**	**2.2 × 10^−7^**
					LEAP versus Chowdhury (Mann–Whitney)	0.0012	**0**.**013**	**0**.**013**
Relative High-alpha PSD	0.10	0.20	0.29	**—**	Difference between groups (Kruskal–Wallis)	**—**	0.13	0.13
					LEAP versus JME (Mann–Whitney)	0.0024	**0**.**042**	0.13
					Chowdhury versus JME (Mann–Whitney)	0.0010	0.73	0.73
					LEAP versus Chowdhury (Mann–Whitney)	0.0014	0.26	0.52
Relative Beta PSD	0.38	0.38	0.22	**—**	Difference between groups (Kruskal–Wallis)	**—**	**2.5 × 10^−5^**	**7.5 × 10^−5^**
					LEAP versus JME (Mann–Whitney)	−0.00014	0.083	0.083
					Chowdhury versus JME (Mann–Whitney)	0.00060	**0**.**00013**	**0**.**0003**
					LEAP versus Chowdhury (Mann–Whitney)	−0.00073	**1.3 × 10^−5^**	**3.9 × 10^−5^**
Peak alpha frequency	**1.3 × 10^−5^**	**—**	0.66	0.67	Difference between groups (Kruskal–Wallis)	**—**	**1.4 × 10^−5^**	**—**
					LEAP versus JME (Mann–Whitney)	0.45	**0**.**0044**	**0**.**0088**
					Chowdhury versus JME (Mann–Whitney)	0.84	**2.1 × 10^−5^**	**6.3 × 10^−5^**
					LEAP versus Chowdhury (Mann–Whitney)	−0.39	**0**.**039**	**0**.**039**
Log_10_ alpha shift	**0**.**00012**	**—**	0.54	0.65	Difference between groups (Kruskal–Wallis)	**—**	**0**.**00059**	**—**
					LEAP versus JME (Mann–Whitney)	−0.21	**0**.**0016**	**0**.**0048**
					Chowdhury versus JME (Mann–Whitney)	−0.24	**0**.**0037**	**0**.**0074**
					LEAP versus Chowdhury (Mann–Whitney)	0.030	0.77	0.77
Mean strength	**0**.**00012**	**0**.**0006**	0.48	0.65	Difference between groups (Kruskal–Wallis)	**—**	**0**.**00022**	**0**.**00066**
					LEAP versus JME (Mann–Whitney)	−0.73	**0**.**00013**	**0**.**00039**
					Chowdhury versus JME (Mann–Whitney)	−0.38	**0**.**039**	0.078
					LEAP versus Chowdhury (Mann–Whitney)	−0.35	0.067	0.078
Mean strength variance	0.15	0.52	0.22	**—**	Difference between groups (Kruskal–Wallis)	**—**	**9.0 × 10^−5^**	**0**.**00037**
					LEAP versus JME (Mann–Whitney)	0.063	0.32	0.32
					Chowdhury versus JME (Mann–Whitney)	−0.80	**0**.**00012**	**0**.**00024**
					LEAP versus Chowdhury (Mann–Whitney)	0.86	**5.6 × 10^−5^**	**0**.**00017**
Clustering coefficient	0.33	0.66	0.10	**—**	Difference between groups (Kruskal–Wallis)	**—**	**2.8 × 10^−7^**	**1.4 × 10^−6^**
					LEAP versus JME (Mann–Whitney)	0.011	**0**.**033**	**0**.**033**
					Chowdhury versus JME (Mann–Whitney)	−0.023	**9.0 × 10^−6^**	**1.8 × 10^−5^**
					LEAP versus Chowdhury (Mann–Whitney)	0.034	**1.0 × 10^−7^**	**3.0 × 10^−^** ^7^
Path length	0.13	0.52	0.17	**—**	Difference between groups (Kruskal–Wallis)	**—**	**0**.**022**	**0**.**044**
					LEAP versus JME (Mann–Whitney)	0.00059	1.0	1.0
					Chowdhury versus JME (Mann–Whitney)	−0.014	**0**.**0071**	**0**.**021**
					LEAP versus Chowdhury (Mann–Whitney)	0.014	**0**.**022**	**0**.**044**
Small-world index	0.61	0.66	0.02	**—**	Difference between groups (Kruskal–Wallis)	—	**0**.**040**	**0**.**044**
					LEAP versus JME (Mann–Whitney)	0.0080	0.074	0.15
					Chowdhury versus JME (Mann–Whitney)	−0.0093	0.19	0.19
					LEAP versus Chowdhury (Mann–Whitney)	0.017	**0**.**013**	**0**.**039**
BNI	**5.6 × 10^−5^**	**—**	0.56	0.65	Difference between groups (Kruskal–Wallis)	**—**	**0**.**00021**	**—**
					LEAP versus JME (Mann–Whitney)	−2.87	**0**.**00021**	**0**.**00063**
					Chowdhury versus JME (Mann–Whitney)	−1.47	**0**.**012**	**0**.**024**
					LEAP versus Chowdhury (Mann–Whitney)	−1.40	0.19	0.19

BNI = brain network ictogenicity; JME = juvenile myoclonic epilepsy; LEAP = Longitudinal European Autism Project; PSD = power spectral density; AUC ROC = Area under the receiver operating curve. Bold text indicates *P* < 0.05.

**Figure 2:**
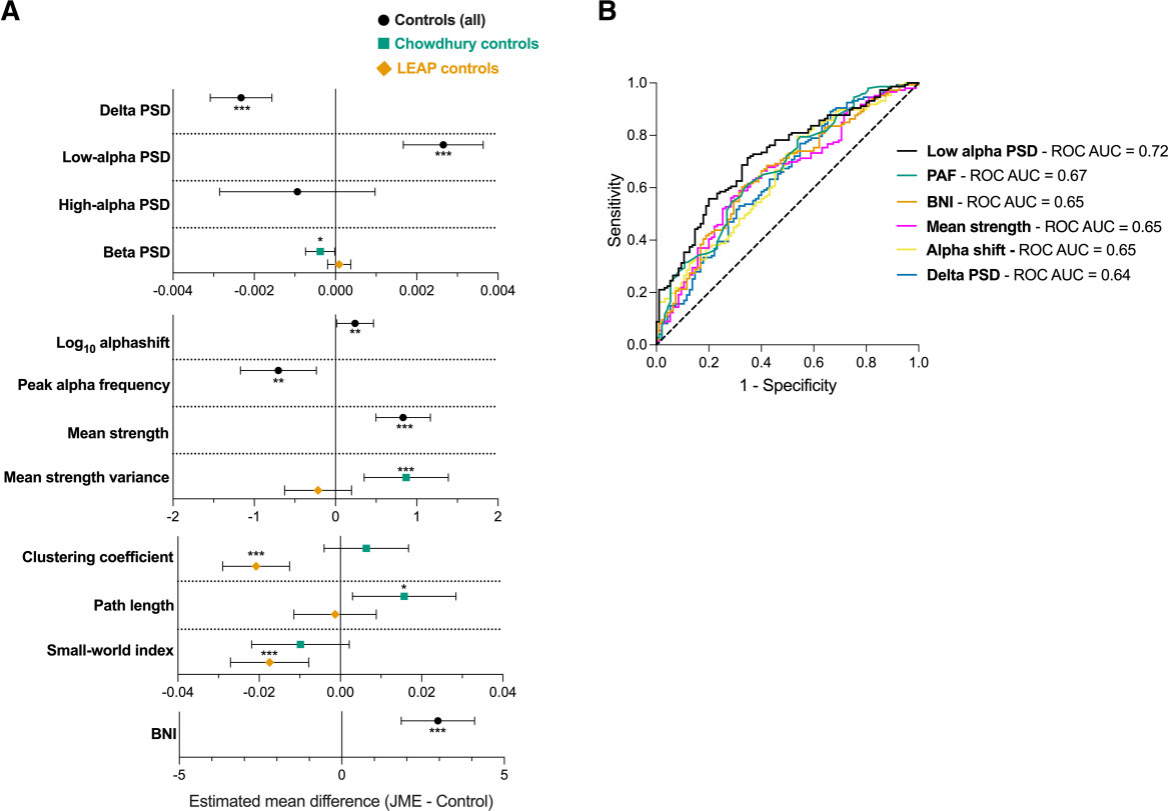
**Results from analysis of EEG features between JME and controls.** (**A**) Summary of the estimated marginal mean difference of EEG features in JME compared with controls, from multiple linear regression models. Models control for epoch length, age (for all measures) and EEG time (for log_10_ alpha shift and PAF only). The central marker shows estimated marginal mean difference and error bars are 95% confidence intervals. Model result tables, including beta coefficients and exact *P*-values are presented in Supplementary material. *P < 0.05, **P < 0.01 and ***P < 0.001. (**B**) ROC curves for EEG features in JME and controls. Area under the ROC values are presented in the legend. Low-alpha PSD, Alpha shift & Delta PSD: JME *N* = 147, control *N* = 95; PAF: JME *N* = 146, control *N* = 93; mean strength and BNI: JME N = 146, control *N* = 95 BNI = brain network ictogenicity; JME = juvenile myoclonic epilepsy; LEAP = Longitudinal European Autism Project; PAF = peak alpha frequency; PSD = power spectral density; ROC AUC = area under the receiver operating curve

To account for different subgroups and the effect of potential confounders (age, sex, epoch length, EEG time), multiple linear regression models were performed for each EEG measure, containing the appropriate covariates. A summary of the differences in EEG features between JME and Controls are presented in Fig. 2A, and all regression models presented in Supplementary Tables 2–6. Relative low-alpha PSD (untreated JME β = 0.0033, *P* = 2.9 × 10^−7^; treated JME β = 0.0022, *P* = 9.1 × 10^−5^) and BNI (β = 3.0, *P* = 5.1 × 10^−7^) were the features with the greatest difference between JME and controls whilst accounting for covariates (Fig. 2A).

### Receiver operating characteristic analysis

To test which EEG feature best served to discriminate JME and control EEGs, ROC curve analysis was performed for delta PSD, low-alpha PSD, PAF, alpha shift, mean strength and BNI, based on the results of the analysis above. Low-alpha PSD had the greatest ROC AUC of 0.72 (95% CI = 0.66–0.79, *P* = 7.2 × 10^−9^), followed by PAF (AUC = 0.67, 95% CI = 0.60–0.74, *P* = 7.4 × 10^−6^) and BNI (AUC = 0.65, 95% CI = 0.58–0.72, *P* = 0.000056) (Fig. 2B).

A proposed cut-off value for low-alpha PSD is >0.0066, which has a sensitivity of 0.69 and a specificity of 0.67 (Supplementary Fig. 4). Using this threshold of relative low-alpha PSD gives a positive predictive value (PPV) of 77% and a negative predictive value (NPV) of 58%.

Using only untreated JME EEGs compared with controls increases the ROC AUC for low-alpha PSD to 0.78 (95% CI = 0.70–0.85, *P* = 6.2 × 10^−9^). The same thresholding method gave the same cut-off value of >0.0066, as above (sensitivity = 75%, specificity = 67%), giving a PPV of 59% and a NPV of 81%.

### Heterogeneity of EEG features within and between cohorts

#### Influence of confounding factors on EEG features

##### Sex

There was a weak association of higher PAF in females with JME (U = 1841, *P* = 0.07), otherwise there were no sex differences in any EEG feature in JME or control groups.

##### Age, epoch length and EEG time

**Figure 3:**
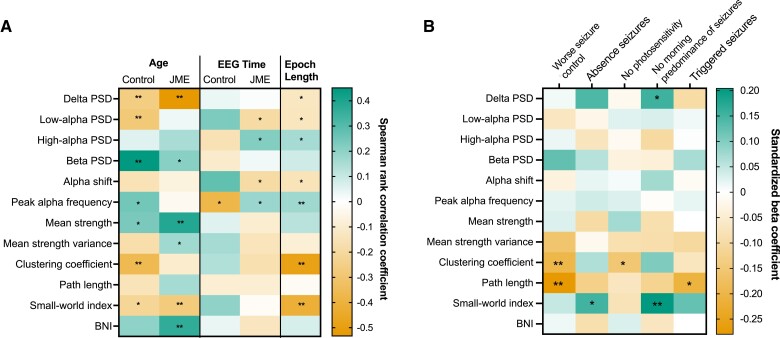
**Factors influencing EEG features within and between cohorts.** (**A**) Heatmap representing the influence of continuous covariates on each EEG feature in control and JME cohorts. Colour represents the Spearman’s rank correlation coefficient between the continuous covariates and EEG features (green = positive correlation, red = negative correlation). Age and EEG time results are stratified by JME/controls. Test statistics, exact *P*-values and N for each correlation is displayed in Supplementary Table 7. (**B**) Heatmap showing the standardized beta coefficients of clinical variables in multiple linear regression models of EEG features in JME cohort. All models control for age and epoch length. Unstandardized and standardized β coefficients and exact *P*-values for each association are displayed in Supplementary Table 8. *P < 0.05, **P < 0.01 BNI = brain network ictogenicity; JME = juvenile myoclonic epilepsy; PPR = photoparoxysmal response; PSD = power spectral density; Sz = seizures

Figure 3A and Supplementary Table 7 shows the results of Spearman’s rank correlation between each EEG feature and potentially confounding continuous variables (age, EEG time and epoch length) in controls and JME. Age and epoch length were associated with a variety of frequency-based and network-based EEG features, whilst EEG time was associated only with frequency-based features. Interestingly, the relationship between PAF and EEG time was opposing in controls (r_s_ = −0.38, *P* = 0.015) and JME (r_s_ = 0.18, *P* = 0.038).

##### Anti-seizure medication treatment

Low-alpha PSD was significantly higher in untreated JME EEGs compared with treated JME EEGs (β = −0.002, *P* = 0.03) in a multiple linear regression model controlling for age. *P*-values for ASM treatment status were ≥0.1 for all other EEG features.

##### EEG site

The site at which BIOUME EEGs were recorded had no significant association (*P* > 0.28) with any frequency-based EEG features. Clustering coefficient was the only feature to show evidence of a difference between sites [ANCOVA: (F(6130) = 2.14, *P* = 0.053), Supplementary Fig. 5]. No other network-based measure significantly differed between sites (*P* > 0.20).

#### Test–retest reliability

Table 5A shows the test–retest reliability between epochs in the same EEG recording and Table 5B between multiple EEG recordings in the same ASM treatment state in JME participants. Between epochs, there is excellent reliability (r_s_ > 0.87, ICC > 0.88) for all frequency-based measures, with differences in epoch length having a minimal effect on reliability. However, for network topology measures there is lower test–retest reliability but improving when epoch length is consistent. BNI has a good reliability (r_s_ > 0.73, ICC > 0.77), with epoch length having little effect.

**Table 5:** (**A**) The test–retest reliability of EEG features in different epochs from the same EEG. ICC (intra-class correlation). (**B**) The test–retest reliability of EEG features in multiple EEG recordings taken when the participant was in the same treatment state (either both in the untreated state or both in the treated state)

(A) Test–retest reliability (between epochs in the same EEG)				
EEG feature	Spearman’s *r*		ICC	
	All epochs (*n* = 178)	Equal length epochs (*n* = 116)	All epochs (95% CI) (*n* = 178)	Equal length epochs (*n* = 116)
Delta PSD	0.90	0.90	0.91 (0.88–0.93)	0.91 (0.88–0.94)
Low-alpha PSD	0.90	0.92	0.91 (0.88–0.93)	0.93 (0.91–0.95)
High-alpha PSD	0.90	0.91	0.88 (0.84–0.91)	0.91 (0.87–0.94)
Beta PSD	0.91	0.95	0.90 (0.87–0.92)	0.94 (0.92–0.96)
Peak alpha frequency	0.88	0.86	0.87 (0.84–0.91)	0.89 (0.85–0.92)
Log_10_ alpha shift	0.91	0.91	0.91 (0.88–0.93)	0.93 (0.90–0.95)
Mean strength	0.79	0.83	0.82 (0.76–0.86)	0.85 (0.79–0.89)
Mean strength variance	0.69	0.74	0.73 (0.66–0.80)	0.78 (0.70–0.84)
Clustering coefficient	0.41	0.36	0.59 (0.48–0.68)	0.64 (0.52–0.74)
Path length	0.45	0.54	0.49 (0.38–0.60)	0.55 (0.41–0.67)
Small-world index	0.44	0.44	0.55 (0.44–0.64)	0.58 (0.45–0.69)
BNI	0.76	0.81	0.78 (0.72–0.83)	0.81 (0.73–0.86)
**(B) Test–retest reliability (between multiple EEGs in same treatment state)**				
**EEG feature**	**Spearman’s *r***		**ICC**	
	**All epochs (*n* = 21)**	**Equal length epochs ± 5s (*n* = 13)**	**All epochs (95% CI) (*n* = 21)**	**Equal length epochs ± 5s (*n* = 13)**
Delta PSD	0.70	0.80	0.83 (0.57–0.93)	0.86 (0.54–0.96)
Low-alpha PSD	0.72	0.59	0.78 (0.48–0.91)	0.73 (0.09–0.92)
High-alpha PSD	0.34	0.45	0.51 (−0.24–0.81)	0.55 (−0.58–0.86)
Beta PSD	0.72	0.70	0.87 (0.68–0.95)	0.89 (0.62–0.97)
Peak alpha frequency	0.34	0.18	0.48 (−0.27–0.79)	0.36 (−1.34–0.81)
Log_10_ alpha shift	0.54	0.43	0.67 (0.22–0.87)	0.62 (−0.34–0.89)
Mean strength	0.74	0.67	0.85 (0.65–0.94)	0.84 (0.51–0.95)
Mean strength variance	0.38	0.03	0.67 (0.18–0.87)	0.55 (−0.59–0.86)
Clustering coefficient	0.18	0.41	0.54 (−0.05–0.81)	0.86 (0.51–0.96)
Path length	0.42	0.42	0.78 (0.45–0.91)	0.77 (0.25–0.93)
Small-world index	0.46	0.63	0.72 (0.29–0.89)	0.86 (0.53–0.96)
BNI	0.64	0.55	0.80 (0.50–0.92)	0.75 (0.24–0.92)

BNI = brain network ictogenicity; JME = juvenile myoclonic epilepsy; PSD = power spectral density.

The results presented in Table 5B should be interpreted with more caution due to the smaller number of EEGs available for this analysis (total *n* = 21) and the potential effect of age on these measures (13 EEGs were performed ≤1 year apart, 8 EEGs between 2 and 10 years apart). To account for epoch length, we further ran the analysis including only epochs ± 5 s difference in length. The reliability is much more variable across the frequency and network topology measures, however, remains good (ICC > 0.75) for delta, low-alpha and beta PSD, mean strength, path length and BNI.

#### Associations of EEG features with clinical variables within juvenile myoclonic epilepsy cohort

Figure 3B shows a summary of associations of each EEG feature with a variety of clinical variables in the JME cohort, showing standardized beta coefficients from multiple linear regression models controlling for age and epoch length. The greatest standardized beta-coefficient changes from 0 exist for functional network topology measures, particularly average clustering coefficient, average characteristic path length and small-world index. All clinical outcomes in Fig. 3B (absence seizures, lack of PPR, no morning predominance of seizures and triggered seizures) have been associated with worse seizure control in this dataset.^28^

Additional sensitivity checks of associations of EEG features with seizure prognosis in a limited dataset of only those with JME who report having used sodium valproate (*n* = 83) are reported in Supplementary Table 9. A similar pattern of results is seen in this smaller sample, with a significant association of decreased path length with worse seizure control (standardized β = −0.24, *P* = 0.04). However, unlike in the full sample, increased relative beta PSD is weakly associated with seizure control in this subsample (standardized β = 0.23, *P* = 0.05).

## Discussion

This study uses a range of EEG features to characterize differences in the interictal EEG of individuals with JME compared with controls and assess their heterogeneity within and between cohorts. Our results support findings from previous studies in IGE,^5,7,17^ showing that the low-alpha frequency range is the most abnormal, with significantly elevated low-alpha PSD and lower peak alpha frequency in JME compared with controls. Furthermore, we show that these findings are reproducible when using EEGs from multiple sites and multiple cohorts of patients, giving them considerable external validity and potential for application in settings outside of research. A novel finding was increased BNI in functional networks derived from JME EEGs, which corroborates the same findings in other epilepsies^18,24,25^ and using other modalities, such as MEG.^27^

Functional network topology measures, such as average clustering coefficient, average characteristic path length and small-word index, were the only features which consistently differ within the JME cohort depending on seizure phenotypes. JME patients with poor seizure control (or risk factors for it) have significantly lower path length and clustering coefficient than those with good seizure control.

In addition, our results highlight that confounding factors such as age and epoch length have a considerable effect on many EEG features and so should be carefully controlled for. These confounding factors may explain the variability of findings from previous studies investigating network topology measures in epilepsy.

### Differences in EEG features between juvenile myoclonic epilepsy and controls

#### Power spectral density measures

ROC analysis showed that low-alpha PSD has ‘acceptable’ clinical discriminatory ability between JME and controls (AUC between 0.7–0.8),^40^ with PAF and BNI falling just below this threshold at 0.67 and 0.65, respectively. However, associated *P*-values show these measures are highly significant in their ability to differentiate above chance. Further investigation of relative low-alpha PSD as a biomarker showed satisfactory sensitivity (69%), specificity (67%), PPV (77%) and NPV (58%) in analysis including all JME individuals, improving when comparing controls to only untreated JME individuals (AUC improvement from 0.72 to 0.78). This suggests low-alpha PSD may have promise as a biomarker of JME; however, this requires replication in independent cohorts. A previous study by Schmidt *et al*.^19^ investigated EEG biomarkers of IGE and found that a local coupling biomarker best classified IGEs from controls, compared with other EEG parameters such as average power across the whole EEG power spectrum, peak alpha frequency and mean degree of functional networks. However, this study did not investigate the low-alpha PSD specifically and investigated all IGE syndromes, not just JME.

Neural oscillations in the alpha frequency band are thought to arise from cortico-thalamic interactions and govern top–down control of cognitive processes and feedback processing from higher-order cortical areas to lower-order visual areas.^10^ However, the exact mechanisms of alpha oscillations are complex and poorly understood. Despite the mechanistic complexity, it is clear that alterations in alpha oscillations are seen in a number of neurological and psychiatric disorders, including dementia, schizophrenia, stroke^41^ and epilepsy. The shift in alpha oscillatory activity from a higher frequency in healthy controls to a lower frequency in epilepsy patients, is particularly relevant to IGE syndromes, since dysfunction in thalamocortical connections are thought to drive the generation of spike-wave discharges and generalized seizures.^11,12^

Delta PSD was lower in JME EEGs compared with controls in both univariate and multivariable analysis. Unlike the alpha frequency band, we are not aware of any previous evidence regarding abnormal delta frequency in patients with IGE, and no differences were reported in patients with temporal lobe epilepsy;^42^ therefore replication would be required for any conclusions to be drawn. Furthermore, in the ROC analysis, delta PSD differentiates between controls and JME to a lesser (weaker) extent than other EEG measures (such as low-alpha PSD and BNI), however still warrants further investigation.

#### Network topology measures

Our results indicate that functional network topology, as derived from activity in the 6–9 Hz frequency range, is inherently different in JME compared with healthy controls, supporting evidence from other studies in IGE.^17,19–22,43^ We can conclude that these differences are not due to ASM treatment as there were no differences in network topology measures between untreated and treated individuals with JME. The mean strength of JME functional networks was significantly higher than controls in both univariate and multivariable analysis, and the variance of mean strength was also significantly higher in JME than Chowdhury controls, replicating results from the original study.^17^ An increase in the mean strength of networks in JME indicates that the EEG signal, and therefore brain oscillatory activity, at each node is synchronized to the activity at other nodes to a higher extent than in controls. However, the increase in mean strength variability also indicates that there may be both abnormally under-connected brain regions as well as over-connected regions. The opposing direction of effect between clustering coefficient and path length in Chowdhury/LEAP control networks compared with JME is unexpected and puzzling, and potential factors influencing this, including differences in age and epoch length, are discussed in the following context.

As well as differences in static network topology, this study also investigated differences in JME functional networks using BNI, a computational framework that uses a dynamic model to characterize the likelihood of brain networks to enter a seizure-like state. The use of dynamic models in epilepsy research, such as BNI, shows promise both in the identification of diagnostic and prognostic biomarkers,^18,19,25,27^ and also in aiding the understanding and modelling the transition of the brain into a seizure state. As hypothesized, this study revealed that individuals with JME have an increased likelihood of brain networks to transition to a seizure state (higher BNI) compared with controls.

### Heterogeneity of EEG features within and between cohorts

#### Associations with age, epoch length and time of day

We investigated how factors such as age, time of day and epoch length influenced EEG features in JME and control cohorts. Epoch length was highly associated with network topology measures, particularly clustering coefficient and small-world index, as well as lower frequency PSD measures, supplementing evidence from previous studies.^33^ Age was also associated with multiple EEG features, as expected from knowledge of EEG and brain network changes through development. For example, delta PSD decreased and beta PSD increased with age in both JME and control cohorts, reflecting known associations of EEG oscillations with maturation.^44,45^

Alpha shift (the ratio of low to high-alpha PSD) was negatively correlated with EEG time (lower values later in the day) in JME but showed no association in controls. A morning predominance of seizures is a hallmark of JME and transcranial magnetic stimulation studies indicate cortical excitability is highest in the morning in patients.^46,47^ Hence, the fact that certain EEG parameters differ in JME throughout the day may reflect a differing seizure threshold. However, this analysis was somewhat limited since most JME EEGs took place in the morning, compared to the afternoon for controls. Matching future studies on EEG time would allow this diurnal change in hyperexcitability in JME and the effect on EEG features to be investigated further.

#### Associations with clinical phenotypes

Our investigation of the association of EEG features with clinical phenotypes in the JME cohort showed little evidence of associations with frequency-based EEG features in main analysis, but some evidence of associations with functional network topology. However, when we included only the subset of individuals exposed to valproate, we found some evidence of an association between increased beta PSD with poor seizure control. This slightly differing result limits any conclusions about this association, and highlights the limitation of the commonly used definitions of seizure control/seizure prognosis, which are discussed in the following context.

Previously, work from Abela *et al*.,^5^ showed associations of alpha oscillatory activity with seizure control in IGE and, however more recent evidence from Pegg *et al*.^7^ showed no difference in PSD between well-controlled and drug-resistant patients with IGE. However, we note differing definitions and methods of defining seizure control/prognosis between studies. EEGs in the present study were obtained from patients at any point during their epilepsy clinical care with a median of 4 years between-EEG acquisition and recruitment into the study (when seizure prognosis was recorded). Conversely, Abela *et al*.^5^ and Pegg *et al*.^7^ recorded EEGs at the time of recruitment. Definitions of seizure control also differ between studies, with the present study and Pegg *et al*.^7^ using the commonly used definition of drug resistance in epilepsy^32^ (continuation of seizures despite at least two ASM trials), whereas Abela *et al*.^5^ defined those with poor seizure control as four or more seizures of any type during the 12 months prior to the study inclusion.

Alterations in functional network topology showed associations with clinical features in JME, particularly clustering coefficient, reflecting a network’s functional segregation, and path length, reflecting a network’s functional integration. Shorter average path length and decreased clustering coefficient were associated with poorer seizure control in this JME cohort. Shorter path length was also associated with experiencing triggered seizures, and decreased clustering coefficient was associated with *not* experiencing PPR, both phenotypes associated with having a worse seizure outcome in this cohort.^28^ A short average path length and low clustering is representative of more random networks, whereby information can pass easily through the network from one node to functionally distinct nodes due to longer range functional connections,^48^ and therefore, speculatively, may have an increased likelihood to synchronize more easily, implying an increased vulnerability to seizures. Indeed, JME networks have been shown to transition to more random network topology during spike-wave discharges, with decreased clustering in theta and beta frequency bands.^49^

Conversely, higher small-world index, a measure derived from CC and PL, was significantly associated with experiencing absence seizures. A higher small-world index indicates networks have more regular, lattice-like structures, with high clustering, but also with long range connections from functionally distinct regions, keeping the average path length short. Given that experiencing absence seizures is strongly associated with poorer seizure outcome,^28,50^ it is surprising that differing network types (random versus ordered) are associated with these two phenotypes. However, several studies of network topology in patients with absence seizures have also shown increased clustering, both in the ictal^51^ and interictal state,^43^ indicating networks with a more ordered, lattice-like topology may be more vulnerable to absence seizures. In addition, a study by Lee *et al*.^22^ showed that individuals with absence epilepsy had the highest small-world index compared with other IGE syndromes, including JME.

Previous literature on functional connectivity in epilepsy is variable because results can differ depending on modality (EEG, MEG, fMRI), functional connectivity measure (PLV, coherence, correlation, and so on) and the frequency band [low-alpha (6–9 Hz), high-alpha (10–11 Hz) or wider bands (2–20 Hz)], therefore making comparisons between the present results and previous evidence challenging. A study from Pegg *et al*.,^21^ using EEG derived functional networks from patients with IGE, showed no differences in network topology between well-controlled (*n* = 19) and drug-resistant patients (*n* = 18) at 6–9 Hz nor 10–11 Hz, using PLV. Potential reasons for this difference in results include sampling differences (IGE versus JME only) and a small sample size in the prior study,^21^ potentially reducing the statistical power to detect the differences apparent in this larger cohort.

### Methodological considerations and limitations

In this study, functional networks were modelled in the 6–9 Hz frequency range only. We chose this band based on the results of our frequency analysis showing that the low-alpha range showed the most significant changes in JME compared to controls, but also on previous evidence from frequency and network studies in IGE showing similar results.^5,17,19,34^

As indicated previously, there are limitations in definitions of seizure prognosis in epilepsy research in general that also apply to this study. We decided to use the internationally agreed definition of drug resistance in epilepsy^32^ (continuation of seizures despite ≥2 appropriate ASM trials) to define poor prognosis, and the internationally agreed definition of seizure freedom^32^ (no seizures for at least one year) as good prognosis. The addition of an intermediate group for those who do not fit into either category was used to maximize the sample and avoid excessive heterogeneity within groups which would likely be present if using binary drug-resistant (yes/no) or binary seizure freedom (yes/no) groups.

### Conclusion

Individuals with JME have increased power of neural oscillatory activity at low-alpha frequencies, along with increased BNI compared with controls, supporting evidence from studies in other epilepsies with considerable external validity. There is encouraging evidence for the use of low-alpha PSD as a biomarker of JME but requires replication in an independent data set. Functional network topology measures are variable and prone to confounding but show significant associations with clinical features and outcomes in JME.

## Supplementary Material

fcac180_Supplementary_Data

## Appendix I

### BIOJUME consortium:

Alessandro Orsini, Alice Howell, Alison Hyde, Alison McQueen, Almu Duran, Alok Gaurav, Amber Collingwood, Amy Kitching, Amy Shakeshaft, Anastasia Papathanasiou, Andrea Clough, Andrew Gribbin, Andrew Swain, Ann Needle, Anna Hall, Anna Smith, Anne Macleod, Asyah Chhibda, Beata Fonferko-Shadrach, Bintou Camara, Boyanka Petrova, Carmel Stuart, Caroline Hamilton, Caroline Peacey, Carolyn Campbell, Catherine Cotter, Catherine Edwards, Catie Picton, Charlotte Busby, Charlotte Quamina, Charlotte Waite, Charlotte West, Ching Ching Ng, Christina Giavasi, Claire Backhouse, Claire Holliday, Claire Mewies, Coleen Thow, Dawn Egginton, Debbie Dickerson, Debbie Rice, Dee Mullan, Deirdre Daly, Dympna Mcaleer, Elena Gardella, Elma Stephen, Eve Irvine, Eve Sacre, Fan Lin, Gail Castle, Graham Mackay, Halima Salim, Hannah Cock, Heather Collier, Helen Cockerill, Helen Navarra, Hilda Mhandu, Holly Crudgington, Imogen Hayes, Ioannis Stavropoulos, Jacqueline Daglish, Jacqueline Smith, Jacqui Bartholomew, Janet Cotta, Javier Peña Ceballos, Jaya Natarajan, Jennifer Crooks, Jennifer Quirk, Jeremy Bland, Jo Sidebottom, Joanna Gesche, Joanne Glenton, Joanne Henry, John Davis, Julie Ball, Kaja K. Selmer, Karen Rhodes, Kelly Holroyd, Kheng Seang Lim, Kirsty O’Brien, Laura Thrasyvoulou, Linetty Makawa, Lisa Charles, Lisa Richardson, Liz Nelson, Lorna Walding, Louise Woodhead, Loveth Ehiorobo, Lynn Hawkins, Lynsey Adams, Margaret Connon, Marie Home, Mark Baker, Mark Mencias, Mark P. Richardson, Mark Sargent, Marte Syvertsen, Matthew Milner, Mayeth Recto, Michael Chang, Michael O'Donoghue, Michael Young, Munni Ray, Naim Panjwani, Naveed Ghaus, Nikil Sudarsan, Nooria Said, Owen Pickrell, Patrick Easton, Paul Frattaroli, Paul McAlinden, Rachel Harrison, Rachel Swingler, Rachel Wane, Rebecca Ramsay, Rikke S. Møller, Robert McDowall, Rosie Clegg, Sal Uka, Sam White, Samantha Truscott, Sarah Francis, Sarah Tittensor, Sarah-Jane Sharman, Seo-Kyung Chung, Shakeelah Patel, Shan Ellawela, Shanaz Begum, Sharon Kempson, Sonia Raj, Sophie Bayley, Stephen Warriner, Susan Kilroy, Susan MacFarlane, Thomas Brown, Tinashe Samakomva, Tonicha Nortcliffe, Verity Calder, Vicky Collins, Vicky Parker, Vivien Richmond, William Stern, Zena Haslam, Zuzana Šobíšková, Amit Agrawal, Amy Whiting, Andrea Pratico, Archana Desurkar, Arun Saraswatula, Bridget MacDonald, Choong Yi Fong, Christoph P. Beier, Danielle Andrade, Darwin Pauldhas, David A. Greenberg, David Deekollu, Deb K. Pal, Dina Jayachandran, Dora Lozsadi, Elizabeth Galizia, Fraser Scott, Guido Rubboli, Heather Angus-Leppan, Inga Talvik, Inyan Takon, Jana Zarubova, Jeanette Koht, Julia Aram, Karen Lanyon, Kate Irwin, Khalid Hamandi, Lap Yeung, Lisa J. Strug, Mark Rees, Markus Reuber, Martin Kirkpatrick, Matthew Taylor, Melissa Maguire, Michalis Koutroumanidis, Muhammad Khan, Nick Moran, Pasquale Striano, Pronab Bala, Rahul Bharat, Rajesh Pandey, Rajiv Mohanraj, Rhys Thomas, Rosemary Belderbos, Seán J. Slaght, Shane Delamont, Shashikiran Sastry, Shyam Mariguddi, Siva Kumar, Sumant Kumar, Tahir Majeed, Uma Jegathasan, William Whitehouse. For details of BIOJUME sites see Supplementary material.

## Abbreviations

ANCOVA =: analysis of covariance
ASM =: anti-seizure medication
AUC =: area under the curve
BIOJUME =: Biology of Juvenile Myoclonic Epilepsy
BNI =: brain network ictogenicity
GTCS =: generalized tonic–clonic seizure
IGE =: idiopathic generalized epilepsy
ICA =: independent component analysis
ICC =: intra-class correlation
IQ =: intelligence quotient
JME =: juvenile myoclonic epilepsy
KCL =: King’s College London
LEAP =: Longitudinal European Autism Project
MEG =: magnetoencephalography
PAF =: peak alpha frequency
PLV =: phase-locking value
PPR =: photoparoxysmal response
PSD =: power spectral density
REDCap =: Research Electronic Data Capture
ROC =: receiver operating characteristic
UCBM =: University Campus BioMedico
UMCU =: University Medical Centre Utrecht
VIF =: variance inflation factor
