# Supplementary material for: Heterogeneity of resting-state EEG features in juvenile myoclonic epilepsy and controls
Source: Brain Commun. 2022 Jul 8;4(4):fcac180. doi: 10.1093/braincomms/fcac180 (PMC9301584; doi:10.1093/braincomms/fcac180)
Supplement: fcac180_Supplementary_Data [file fcac180_supplementary_data.zip › Supplementary_material 1.pdf]

Appendix

Sites and site investigators in the BIOJUME consortium.

Country	Site	Site principal investigators (PIs) and research staff
Canada	SickKids Hospital, Toronto	Lisa Strug (PI), Naim Panjwani, Fan Lin
	Toronto Western Hospital	Danielle Andrade (PI)
Czech Republic	Charles University	Jana Zarubova (PI), Zuzana Šobíšková
Denmark	Danish National Epilepsy Centre	Guido Rubboli (PI), Rikke S. Møller, Elena Gardella
	Syddansk Universitet	Christoph P. Beier (PI), Joanna Gesche
Estonia	Tallinn Children's Hospital	Inga Talvik (PI)
Italy	Commissione Genetica	Pasquale Striano (PI), Alessandro Orsini
	University of Catania	Andrea Pratico (PI)
Malaysia	University of Malaya	Choong Yi Fong (PI), Ching Ching Ng, Kheng Seang Lim
Norway	Vestre Viken Health Trust	Jeanette Koht, Kaja K. Selmer, Marte Syvertsen (Co-PIs)
UK	Airedale NHS Foundation Trust	Pronab Bala (PI), Amy Kitching
	Ashford and St. Peter's Hospitals NHS Foundation Trust	Kate Irwin (PI), Lorna Walding, Lynsey Adams
	Bradford Teaching Hospitals NHS Foundation Trust	Uma Jegathasan (PI), Rachel Swingler, Rachel Wane
	Brighton and Sussex University Hospitals NHS Trust	Julia Aram (Co-PI), Nikil Sudarsan (Co-PI), Dee Mullan, Rebecca Ramsay, Vivien Richmond, Mark Sargent, Paul Frattaroli
	Calderdale and Huddersfield Foundation Trust	Matthew Taylor (PI), Marie Home, Sal Uka, Susan Kilroy, Tonicha Nortcliffe, Halima Salim, Kelly Holroyd
	Cardiff & Vale University Health Board	Khalid Hamandi (PI), Alison McQueen, Dympna Mcaleer
	County Durham and Darlington NHS Foundation Trust	Dina Jayachandran (PI), Dawn Egginton,
	Croydon Health Services NHS Trust	Bridget MacDonald (PI), Michael Chang
	Cwm Taf Morgannwg University Health Board	David Deekollu (Co-PI), Alok Gaurav (Co-PI), Caroline Hamilton, Jaya Natarajan
	Dartford and Gravesham NHS Trust	Shane Delamont (PI), Carmel Stuart, Imogen Hayes
	East and North Hertfordshire NHS Trust	Inyan Takon (PI), Janet Cotta
	East Kent Hospitals University NHS Foundation Trust	Nick Moran (PI), Jeremy Bland
	East Lancashire Hospitals NHS Trust	Rosemary Belderbos (PI), Heather Collier, Joanne Henry, Matthew Milner, Sam White
	Guy's and St Thomas' NHS Foundation Trust	Michalis Koutroumanidis (PI), Javier Peña Ceballos, William Stern
	King's College Hospital NHS Foundation Trust	Mark P. Richardson (Co-PI), Jennifer Quirk (Co-PI), Javier Peña Ceballos, Anastasia Papathanasiou
	King's College London	Deb K. Pal (PI), Mark P. Richardson, Holly Crudgington, Anna Hall, Amber Collingwood, Amy Shakeshaft, Ioannis Stavropoulos, Anna Smith, Robert McDowall, Sophie Bayley
	Kingston Hospital NHS Foundation Trust	Dora Lozsadi (PI), Andrew Swain, Charlotte Quamina, Jennifer Crooks
	Lancashire Teaching Hospitals NHS Foundation Trust	Tahir Majeed (PI), Sonia Raj, Shakeelah Patel, Michael Young
	Leeds Teaching Hospitals NHS Trust	Melissa Maguire (Co-PI), Munni Ray (Co-PI), Caroline Peacey, Linetty Makawa, Asyah Chhibda, Eve Sacre, Shanaz Begum
	Manchester University NHS Foundation Trust	Lap Yeung (Co-PI), Claire Holliday, Louise Woodhead, Karen Rhodes
	Newcastle upon Tyne Hospitals NHS Foundation Trust	Rhys Thomas (Co-PI), Shan Ellawela (Co-PI), Joanne Glenton, Verity Calder, John Davis, Paul McAlinden, Sarah Francis

	NHS Grampian	Karen Lanyon (Co-PI), Graham Mackay (Co-PI), Elma Stephen (Co-PI), Coleen Thow, Margaret Connon
	NHS Tayside	Martin Kirkpatrick (PI), Susan MacFarlane, Anne Macleod, Debbie Rice
	North Tees and Hartlepool NHS Foundation Trust	Siva Kumar (PI), Carolyn Campbell, Vicky Collins
	Nottingham University Hospitals NHS Trust	William Whitehouse (PI), Christina Giavasi (PI), Boyanka Petrova, Thomas Brown, Catie Picton, Michael O'Donoghue, Charlotte West, Helen Navarra
	Portsmouth Hospitals NHS Trust	Seán J. Slaght (PI), Catherine Edwards, Andrew Gribbin, Liz Nelson, Stephen Warriner
	Royal Free London NHS Foundation Trust	Heather Angus-Leppan (PI), Loveth Ehiorobo, Bintou Camara, Tinashe Samakomva
	Salford Royal NHS Foundation Trust	Rajiv Mohanraj (PI), Vicky Parker
	Sandwell & West Birmingham Hospitals NHS Trust	Rajesh Pandey (PI), Lisa Charles, Catherine Cotter
	Sheffield Children's NHS Foundation Trust	Archana Desurkar (PI), Alison Hyde, Rachel Harrison
	Sheffield Teaching Hospitals NHS Foundation Trust	Markus Reuber (PI), Rosie Clegg, Jo Sidebottom, Mayeth Recto, Patrick Easton, Charlotte Waite, Alice Howell, Jacqueline Smith, Rosie Clegg
	Southport and Ormskirk Hospital NHS Trust	Shyam Mariguddi (PI), Zena Haslam
	St George's University Hospitals NHS Foundation Trust	Elizabeth Galizia (PI), Hannah Cock, Mark Mencias, Samantha Truscott, Deirdre Daly, Hilda Mhandu, Nooria Said
	Swansea University Medical School and Swansea Bay University Healthboard	Mark Rees (PI), Seo-Kyung Chung, Owen Pickrell, Beata Fonferko-Shadrach, Mark Baker
	Taunton & Somerset NHS Foundation Trust	Amy Whiting (PI), Kirsty O'Brien
	The Mid Yorkshire Hospitals NHS Trust	Fraser Scott (Co-PI), Naveed Ghaus (Co-PI), Gail Castle, Jacqui Bartholomew, Ann Needle, Julie Ball, Andrea Clough
	The Royal Wolverhampton NHS Trust	Shashikiran Sastry (PI), Charlotte Busby
	The Walton Centre NHS Foundation Trust	Amit Agrawal (PI), Debbie Dickerson, Almu Duran
	University Hospitals Birmingham NHS Foundation Trust	Muhammad Khan (PI), Laura Thrasyvoulou, Eve Irvine, Sarah Tittensor, Jacqueline Daglish
	University Hospitals of Derby and Burton NHS Foundation Trust	Sumant Kumar (PI), Claire Backhouse, Claire Mewies
	University Hospitals Plymouth NHS Trust	Rahul Bharat (PI), Sarah-Jane Sharman
	Walsall Healthcare NHS Trust	Darwin Pauldhas (PI), Sharon Kempson, Lisa Richardson, Lynn Hawkins
	West Suffolk NHS Foundation Trust	Arun Saraswatula (PI), Helen Cockerill
USA	Nationwide Children's Hospital, Ohio	David A. Greenberg (PI)
