# Supplementary material for: Heterogeneity of resting-state EEG features in juvenile myoclonic epilepsy and controls
Source: Brain Commun. 2022 Jul 8;4(4):fcac180. doi: 10.1093/braincomms/fcac180 (PMC9301584; doi:10.1093/braincomms/fcac180)
Supplement: fcac180_Supplementary_Data [file fcac180_supplementary_data.zip › Supplementary_Material 2.pdf]

Supplementary Material

Supplementary methods

Power spectral density (PSD) and frequency measures

All PSD and frequency analysis was carried out using Fieldtrip or custom written MATLAB scripts. To remove any remaining artifacts in the data between 2-20Hz (e.g. eye or cardiac artifacts), independent component analysis (ICA) was carried out using Fieldtrip. Where ICA components or channels with high amounts of noise were removed, data were re-referenced to average.

The MATLAB p-welch function (using a 1-second Hanning window with 50% overlap) was used to perform the Fast Fourier Transform to provide the PSD from each epoch. The frequency range 2-20Hz was selected as it encapsulates frequency bands commonly used in the literature, while avoiding low-frequency drifts (<2 Hz), as well as high frequency muscle artefacts (>20 Hz). The PSD between 2-20Hz, at 0.1Hz increments, was then normalised against the total power of each EEG channel to give the relative power. The mean relative power across all channels was then calculated for each EEG.

Data-driven frequency bands from Shackman et al.[1] were used (delta: 2-5Hz, alpha-low: 6-9Hz, alpha-high: 10-11Hz, beta: 12-19Hz, and gamma: >21Hz). The lower limit of the delta frequency range was set to 2Hz and the gamma frequency range was excluded due to our previous selection of data in the 2-20Hz range only. The mean relative power in each frequency band was then calculated.

Alpha Shift

The shift in alpha power from high to low-alpha was calculated following methods in Abela et al.[2] Relative power in the low-alpha frequency band (6-9Hz) was divided by relative power in the high-alpha frequency band (10-11Hz) to give the alpha shift. To normalise the distribution of this variable the log_{10} alpha shift was used for analysis.

Peak alpha frequency

To extract the peak alpha frequency from each EEG power spectrum, the 1/f background noise from the power spectrum was first removed to ensure that only oscillatory activity was being measured, based on methodology from Haller et al.[3] This involved using the robust regression function in MATLAB to fit a line to the power spectrum in semi-log space, but to avoid the alpha region (6-13Hz) when fitting the line so that the alpha oscillatory signal did not bias the fit. The background noise underneath the line was then removed from the spectrum. From this, the MATLAB 'findpeaks' function was used to find the largest peak in the remaining oscillatory spectrum between 2 to 20Hz. The frequency at which this peak existed was then identified as the peak alpha frequency for each individual epoch.

Inferring functional networks from EEG

Inferring of functional networks uses methods described in Lopes et al.[4] To summarise, functional networks were calculated using phase-locking value (PLV)[5-9] on EEG epochs which had been Butterworth bandpass filtered between 6-9Hz. PLV was chosen due to its ability to differentiate IGE from controls.[8,9] Network nodes were electrode locations and PLV values as network edges/connectivity weights. For each pair of nodes i and j,

$$PLV_{ij} = \frac{1}{N_s}\left|\sum_{k=1}^{N_s} e^{i\Delta\phi_{ij}(t_k)}\right|,$$

where N_s is the number of samples, and $\Delta\phi_{ij}(t_k)$ is the instantaneous phase difference between the signals recorded from electrodes i and j at time t_k, computed using the Hilbert transform. We also found the average phase-lag τ_{ij} between the two signals,

$$\tau_{ij} = arg\left(\sum_{k=1}^{N_s} e^{i\Delta\phi_{ij}(t_k)}\right).$$

Nodes i and j were considered connected if PLV_{ij} >0 and τ_{ij}>0. Non-zero time-lag PLV was used to avoid false signal connections due to volume conduction.[10] 99 surrogate networks were generated from the original EEG signals using the iterative amplitude-adjusted Fourier transform with 10 iterations.[11,12] Connections were replaced with 0 if their PLV_{ij} weights were below the 95% significance level compared to the same connection weights as computed from the surrogates. This method gave a directed, weighted functional network a_{ij} from each EEG epoch.

Network topology measures

The methodology used to characterise the functional networks was adapted from Chowdhury et al.[13] to account for our networks being weighted and directed. We used Brain Connectivity Toolbox (http://www.brain-connectivity-toolbox.net/) to calculate mean strength (the same measure is referred to as mean degree in Chowdhury et al.[13]), the variance of the strength distribution of all network nodes, average clustering coefficient and average characteristic path length of each functional network. Since networks were weighted and directed, mean strength is the mean of the sum of in- (a_{ji}) and out- (a_{ij}) strengths to nodes:

$$k_i^{out} = \Sigma_{j\in N} a_{ij}$$

$$k_i^{in} = \Sigma_{j\in N} a_{ji}$$

$$k_i^{total} = k_i^{in} + k_i^{out},$$

whereby N is the set of all nodes.

Average clustering coefficient (CC) here, is average 'intensity' (geometric mean) of all triangles associated with each node (i):

$$t_i = \frac{1}{2}\Sigma_{j,h\in N}(a_{ij} + a_{ji})(a_{ih} + a_{hi})(a_{jh} + a_{hj})$$

$$CC^{original} = \frac{1}{n}\Sigma_{j\in N} \frac{t_i}{(k_i^{out}+k_i^{in})(k_i^{out}+k_i^{in}-1)-2\Sigma_{j\in N} a_{ij}a_{ji}}.$$

For average characteristic path length (PL) (using Floyd-Warshall algorithm), the distance between nodes is the inverse connection strength (1/connection strength) and the average path length is the average shortest path length in the network (whereby g_{ij} is the directed shortest path from i to j and n is the number of nodes):

$$d_{ij} = \Sigma_{a_{ij}\in g_{ij}} a_{ij}$$

$$PL^{original} = \frac{1}{n}\Sigma_{j\in N} \frac{\Sigma_{j\in N, j\neq i} d_{ij}}{n-1}.$$

Because CC and PL are both sensitive to mean degree and strength, these measures were normalized to the mean CC and PL of 500 random surrogate networks with the same distribution of edge weights:

$$CC^{norm} = \frac{CC^{original}}{CC^{surrogate}},$$

$$PL^{norm} = \frac{PL^{original}}{PL^{surrogate}}.$$

The small-world index of each network was also calculated according to Humphries and Gurney [14]:

$$S = \frac{CC^{norm}}{PL^{norm}}.$$

Brain Network Ictogenicity (BNI)

Mathematical model

A theta model was implemented at each network node to model seizure dynamics.[8,15-17] The brain activity at node i was represented by a phase oscillator θ_i. 'Resting-state' is defined as a phase close to a fixed stable phase $\theta^{(s)}$ and an 'oscillatory state' as a rotating phase. The resting-state represents normal brain activity, whereas the oscillatory state depicts seizure-like activity. Nodes can transition between these states through a saddle-node on invariant circle (SNIC) bifurcation. The phase oscillator obeys the following ordinary differential equation:

$$\dot{\theta}_i = (1 - \cos\theta_i) + (1 + \cos\theta_i)I_i(t),$$

where $I_i(t)$ is the input current of node i including noisy inputs and the interaction with the other nodes:

$$I_i(t) = I_0 + \xi^{(i)}(t) + \frac{K}{N}\sum_{j\neq i} a_{ji}\left[1 - \cos\left(\theta_j - \theta^{(s)}\right)\right],$$

where $I_0 + \xi^{(i)}(t)$ is noise, N is the number of nodes, a_{ji} is j, i^{th} entry of the adjacency matrix that encodes the functional network, K is a global scaling factor of the functional network, and $\theta^{(s)}$ is the steady state of the in-neighbor j. The noisy inputs ($I_0 + \xi^{(i)}(t)$) represent signals from other areas of the brain outside of the functional network under consideration, which is assumed to follow a Gaussian distribution (with mean $I_0 = -1.2$ and standard deviation $\sigma^2 = 0.6$, according to previous work[4,16]). Because this study is interested in the influence of functional networks on seizure activity, the chosen parameters ensure that nodes are typically in the resting-state and the transition to seizure-like activity is a result of the network interactions. Lopes et al.[16] showed that different choices of I_0 and σ would not quantitatively change the results. The remaining free value, K, will be discussed below.

BNI measure

BNI[15-17] aims to quantify the propensity of a network to generate seizure-like dynamics in silico. It quantifies the model-generated dynamics with the average fraction of time each node spends in the oscillatory/seizure state:

$$BNI^* = \frac{1}{N}\sum_i \frac{t_{sz}^{(i)}}{T},$$

where $t_{sz}^{(i)}$ is the time that node i spent in the oscillatory state during a total simulation time T. We used T = 4x10^6 time steps and the oscillatory state was defined as any activity larger than a threshold as described in Lopes et al.[16] The time spent in seizure state ($t_{sz}^{(i)}$) depends on the global connectivity strength, K, and therefore so does the BNI.[15,16] To avoid an arbitrary choice of K, we followed the approach described in Lopes et al.[18] where we redefined BNI as:

$$BNI = \int_{K_{min}}^{K_{max}} BNI^*(K)dK,$$

where K_{min} corresponds to the minimum K value for which $BNI^* = 0$ across all computed networks and similarly $K_{max}$to the maximum K value that $BNI^* = 1$. The $[K_{min}, K_{max}]$ interval contained 40 equally distributed global scaling (K) values. For each of these values we computed BNI and therefore obtained a distribution of BNI^* values that results in a curve for each EEG (Supplementary Figure 1). This approach allows for comparison between individuals and avoids arbitrary choice of K. To quantify the variation of BNI^* between K_{min} and K_{max}, the area under the BNI curve generated for each EEG epoch was calculated using the MATLAB 'trapz' function which performs numerical integration via the trapezoidal method.

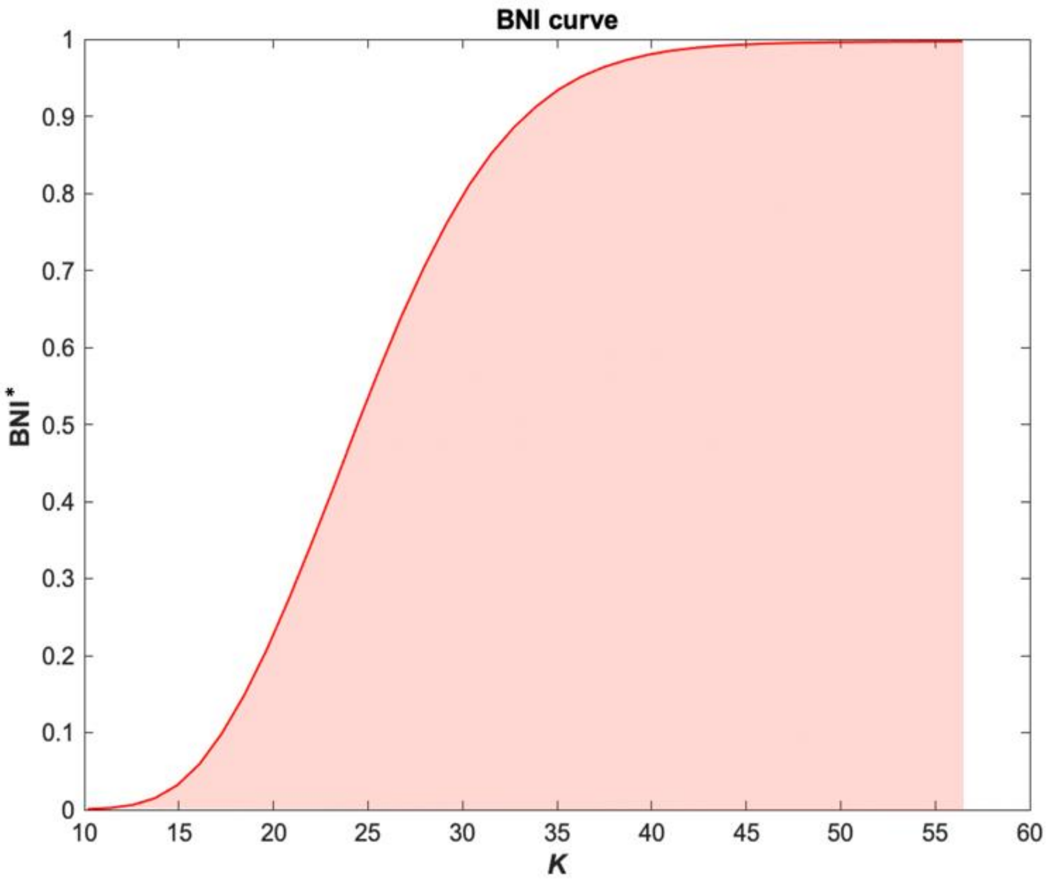

Supplementary Figure 1 - Example Brain Network Ictogenicity (BNI) curve generated by calculating the BNI of a single functional network.

References

1. Shackman AJ, McMenamin BW, Maxwell JS, Greischar LL, Davidson RJ. Identifying robust and sensitive frequency bands for interrogating neural oscillations. *Neuroimage*. 2010;51(4):1319-1333. doi: 10.1016/j.neuroimage.2010.03.037

2. Abela E, Pawley AD, Tangwiriyasakul C, et al. Slower alpha rhythm associates with poorer seizure control in epilepsy. *Ann Clin Transl Neurol*. 2019;6(2):333-343. doi: 10.1002/acn3.710

3. Haller M, Donoghue T, Peterson E, et al. Parameterizing neural power spectra. *bioRxiv*. 2018:299859. doi: 10.1101/299859

4. Lopes MA, Perani S, Yaakub SN, Richardson MP, Goodfellow M, Terry JR. Revealing epilepsy type using a computational analysis of interictal EEG. *Sci Rep*. 2019;9(1):10169. doi: 10.1038/s41598-019-46633-7

5. Lachaux JP, Rodriguez E, Martinerie J, Varela FJ. Measuring phase synchrony in brain signals. *Hum Brain Mapp*. 1999;8(4):194-208. doi: 10.1002/(sici)1097-0193(1999)8:4<194::aid-hbm4>3.0.co;2-c

6. Mormann F, Lehnertz K, David P, E. Elger C. Mean phase coherence as a measure for phase synchronization and its application to the EEG of epilepsy patients. *Physica D: Nonlinear Phenomena*. 2000;144(3):358-369. doi: 10.1016/S0167-2789(00)00087-7

7. Tass P, Rosenblum MG, Weule J, et al. Detection of n:m Phase Locking from Noisy Data: Application to Magnetoencephalography. *Physical Review Letters*. 1998;81(15):3291-3294. doi: 10.1103/PhysRevLett.81.3291

8. Schmidt H, Petkov G, Richardson MP, Terry JR. Dynamics on networks: the role of local dynamics and global networks on the emergence of hypersynchronous neural activity. *PLoS Comput Biol*. 2014;10(11):e1003947. doi: 10.1371/journal.pcbi.1003947

9. Schmidt H, Woldman W, Goodfellow M, et al. A computational biomarker of idiopathic generalized epilepsy from resting state EEG. *Epilepsia*. 2016;57(10):e200-e204. doi: 10.1111/epi.13481

10. Bastos AM, Schoffelen JM. A Tutorial Review of Functional Connectivity Analysis Methods and Their Interpretational Pitfalls. *Front Syst Neurosci*. 2015;9:175. doi: 10.3389/fnsys.2015.00175

11. Schreiber T, Schmitz A. Improved Surrogate Data for Nonlinearity Tests. *Physical Review Letters*. 1996;77(4):635-638. doi: 10.1103/PhysRevLett.77.635

12. Schreiber T, Schmitz A. Surrogate time series. *Physica D: Nonlinear Phenomena*. 2000;142(3):346-382. doi: 10.1016/S0167-2789(00)00043-9

13. Chowdhury FA, Woldman W, FitzGerald TH, et al. Revealing a brain network endophenotype in families with idiopathic generalised epilepsy. *PLoS One*. 2014;9(10):e110136. doi: 10.1371/journal.pone.0110136

14. Humphries MD, Gurney K. Network 'small-world-ness': a quantitative method for determining canonical network equivalence. *PLoS One*. 2008;3(4):e0002051. doi: 10.1371/journal.pone.0002051

15. Goodfellow M, Rummel C, Abela E, Richardson MP, Schindler K, Terry JR. Estimation of brain network ictogenicity predicts outcome from epilepsy surgery. *Sci Rep*. 2016;6:29215. doi: 10.1038/srep29215

16. Lopes MA, Richardson MP, Abela E, et al. An optimal strategy for epilepsy surgery: Disruption of the rich-club? *PLoS Comput Biol*. 2017;13(8):e1005637. doi: 10.1371/journal.pcbi.1005637

17. Petkov G, Goodfellow M, Richardson MP, Terry JR. A critical role for network structure in seizure onset: a computational modeling approach. *Front Neurol*. 2014;5:261. doi: 10.3389/fneur.2014.00261

18. Lopes MA, Richardson MP, Abela E, et al. Elevated Ictal Brain Network Ictogenicity Enables Prediction of Optimal Seizure Control. *Front Neurol*. 2018;9:98. doi: 10.3389/fneur.2018.00098

19. Silvennoinen K, de Lange N, Zagaglia S, et al. Comparative effectiveness of antiepileptic drugs in juvenile myoclonic epilepsy. *Epilepsia Open*. 2019;4(3):420-430. doi: 10.1002/epi4.12349

Supplementary Figures & Tables

Influence of ICA on EEG frequency measures

ICA was used to remove artefacts in 39/95 (41%) of control EEGs and 86/194 (44%) of JME EEGs. There was extremely high correlation between relative PSD in each frequency band in epochs before and after they underwent artifact removal ICA (Supplementary Figure 2 & Supplementary Table 1).

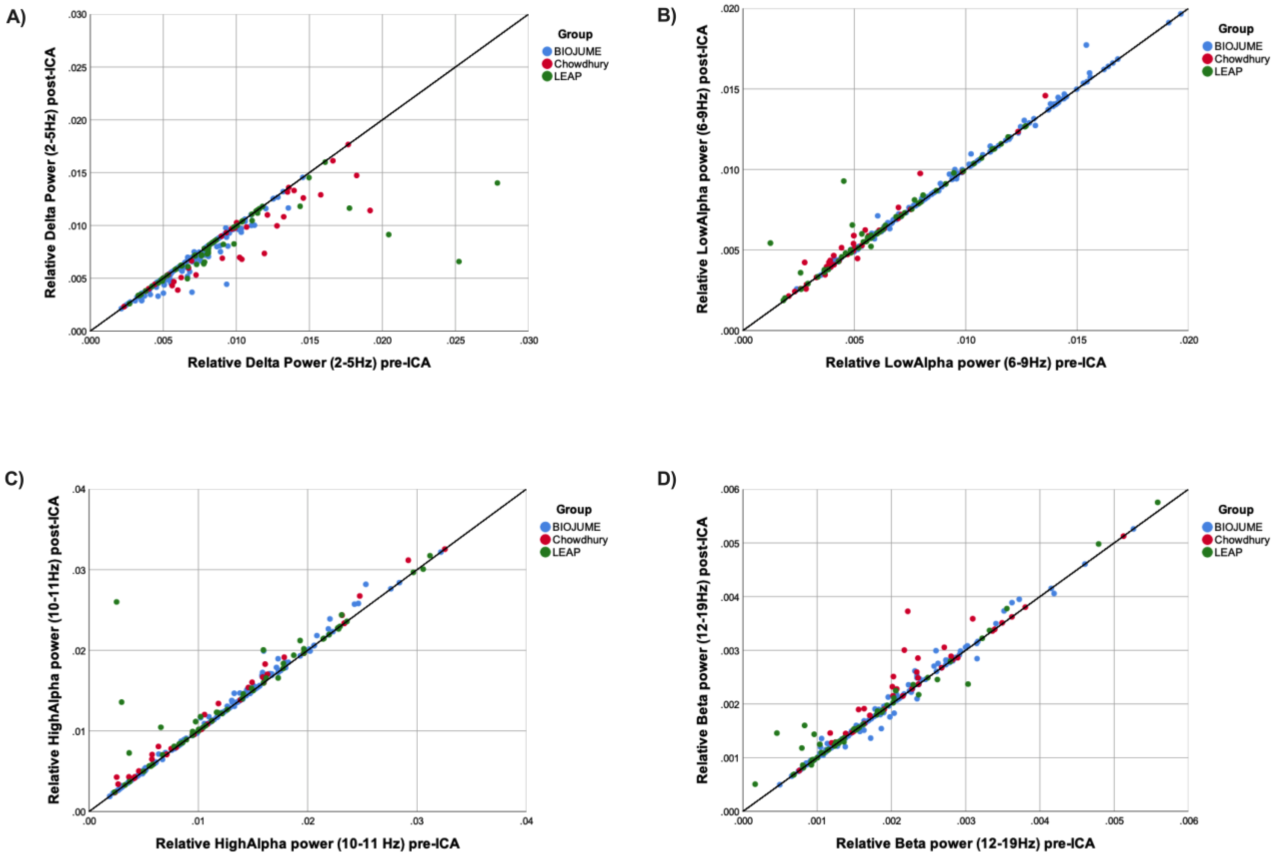

Supplementary Figure 2 - Relative power spectral density measures in **A**) Delta, **B**) Low-alpha **C**) High-alpha and **D**) Beta, pre- and post-independent component analysis (ICA).

Supplementary Table 1 - Pearson's correlation coefficients for pre- and post-ICA relative power spectral density in each frequency band, for each EEG group.

EEG Group	Delta (2-5Hz)	Low-alpha (6-9Hz)	High-alpha (10-11Hz)	Beta (12-19Hz)
**BIOJUME**	0.97	1.0	1.0	0.99
**LEAP**	0.73	0.95	0.89	0.98
**Chowdhury**	0.93	0.98	1.0	0.95

Power spectral density in JME vs Controls

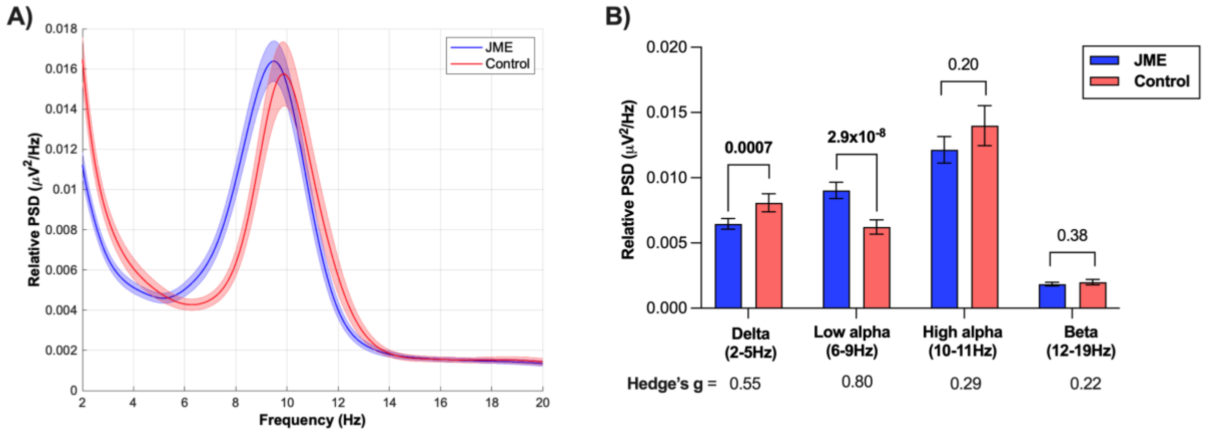

Supplementary Figure 3 - A) Relative power spectral density (PSD) plots of JME and control EEGs between 2-20Hz. Solid line is the subgroup mean and shaded area represents 95% confidence intervals. **B)** Quantification of relative PSD in EEG frequency bands between control and JME EEGs. Values above bars represent p-values from Mann-Whitney U test, corrected for the 4 frequency bands using Bonferroni-holm. Hedge's g values for each comparison are below bars. JME N= 147, Control N = 95. Mann-Whitney U values: Delta PSD =5030, Low-alpha = 3905, High-alpha = 6112, Beta = 6514.

Regression models

Supplementary Table 2 - Multiple linear regression model results for **A)** relative delta power spectral density (PSD), **B)** relative low-alpha PSD, **C)** relative high-alpha PSD and **D)** relative beta PSD. Bold p-values = <0.05. For binary variables categories are presented as they were coded (0/1). Variance inflation factor (VIF) is also reported for each variable.

A) Delta

Variables	Unstandardized coefficient		T	P-value	VIF
	B	Std Error			
Control/JME	-0.0023	0.00039	-6.04	**$6.0x10^{-9}$**	1.17
Age	-0.0001	$2.1x10^{-5}$	-4.67	**$5.0x10^{-6}$**	1.07
Epoch length	-0.00014	$5.1x10^{-5}$	-2.70	**0.0076**	1.21

N =236, adjusted r^2 =0.19

B) Low-alpha

Variables	Unstandardized coefficient		T	P-value	VIF
	B	Std Error			
Control/ Untreated JME	0.0033	0.00062	5.29	**$2.9x10^{-7}$**	1.47
Control/ Treated JME	0.0022	0.00056	3.99	**$9.1x10^{-5}$**	1.31
Age	$-1.6x10^{-5}$	0.000028	-0.99	0.57	1.17
Epoch length	$-2.3x10^{-5}$	0.000067	-0.34	0.73	1.22

N =225, adjusted r^2 =0.15

C) High-alpha

Variables	Unstandardized coefficient		T	P-value	VIF
	B	Std Error			
Age	$8.3x10^{-5}$	$5.3x10^{-5}$	1.56	0.12	1.07
Epoch length	0.00017	0.00013	1.32	0.19	1.21
Control/JME	-0.00094	0.00097	-0.97	0.33	1.17

N =236, adjusted r^2 =0.02

D) Beta

Variables	Unstandardized coefficient		T	P-value	VIF
	B	Std Error			
Age	$2.4x10^{-5}$	$8.0x10^{-5}$	3.09	**0.0023**	1.30
JME/Chowdhury	0.00038	0.00018	2.06	**0.041**	1.39
JME/LEAP	$-9.0x10^{-5}$	0.00014	-0.63	0.53	1.17
Epoch length	$-4.9x10^{-6}$	$1.7x10^{-5}$	-0.29	0.77	1.21

N =236, adjusted r^2 =0.09

Supplementary Table 3 - Results of a multiple linear regression model of log_{10} alpha shift. Bold p-values = <0.05. Control/JME coded as 0/1. Variance inflation factor (VIF) is also reported for each variable. Note high VIF is only present for interaction variables.

Log_{10} Alpha Shift

Variables	Unstandardized coefficient.		T	P value	VIF
	B	St Error			
Control/JME	1.28	0.48	2.66	**0.009**	**41.91**
EEG time* Control/JME	$-2.4x10^{-8}$	$9.7x10^{-9}$	-2.5	**0.013**	**30.06**
Age	-0.006	0.005	-1.38	0.17	1.09
EEG time	$1.1x10^{-8}$	$8.0x10^{-9}$	1.35	0.18	5.09
Mean epoch length	0.0002	0.0083	0.03	0.98	1.12

N =172, adjusted r^2 =0.05

Supplementary Table 4 - Multiple linear regression model of peak alpha frequency. Bold p-values= <0.05. Control/JME coded as 0/1. Variance inflation factor (VIF) is also reported for each variable. Note high VIF is only present for interaction variables.

Peak alpha frequency

Variables	Unstandardized coefficient.		T	P value	VIF
	B	St Error			
Control/JME	-3.18	1.00	-3.18	**0.0018**	42.03
EEG time* Control/JME	$5.8x10^{-5}$	2.0x10-5	2.86	**0.0048**	30.36
EEG time	$-3.7x10^{-5}$	1.7x10-5	-2.24	**0.026**	5.00
Sex	0.24	0.14	1.77	0.078	1.07
Age	0.0012	0.0095	0.12	0.90	1.10
Epoch length	0.005	0.018	-0.03	0.98	1.13

N =168, adjusted r^2 =0.06

Supplementary Table 5 - Results of multiple linear regression analysis of **A)** mean strength, **B)** Mean strength variance, **C)** clustering coefficient, **D)** Path length and **E)** Small-world index of functional networks derived from EEGs. For binary variables categories are presented as they were coded (0/1). Variance inflation factor (VIF) is also reported for each variable.

A) Mean strength

Variables	Unstandardized coefficient.		T	P value	VIF
	B	St Error			
Control/JME	0.832	0.17	4.89	**$1.9x10^{-6}$**	1.17
Epoch length	0.068	0.022	2.96	**0.003**	1.21
Age	0.027	0.009	2.84	**0.005**	1.08

N =235, adjusted r^2 =0.12

B) Mean strength variance

Variables	Unstandardized coefficient.		T	P value	VIF
	B	St Error			
JME/Chowdhury	-1.15	0.26	-4.31	**$2.4x10^{-5}$**	1.40
Age	0.02	0.012	2.25	**0.025**	1.30
Epoch length	-0.02	0.023	-0.89	0.38	1.22
JME/LEAP	0.16	0.2	0.82	0.41	1.18

N =229, adjusted r^2 =0.08

C) Clustering coefficient

Variables	Unstandardized coefficient.		T	P value	VIF
	B	St Error			
Epoch length	-0.004	0.0005	-8.6	**$1.3x10^{-15}$**	1.20
JME/LEAP	0.021	0.004	4.97	**$1.3x10^{-6}$**	1.17
JME/Chowdhury	-0.0064	0.005	-1.21	0.23	1.39
Age	-0.0001	0.0002	-0.47	0.64	1.29

N = 233, adjusted r^2 =0.33

D) Path Length

Variables	Unstandardized coefficient.		T	P value	VIF
	B	St Error			
JME/Chowdhury	-0.061	0.0065	-2.43	**0.016**	1.41
Epoch length	0.00039	0.00059	0.66	0.51	1.21
Age	0.00018	0.00027	0.66	0.51	1.31
JME/LEAP	0.0013	0.0051	0.25	0.80	1.17

N =231, adjusted r^2 =0.01

E) Small-world index

Variables	Unstandardized coefficient.		T	P value	VIF
	B	St Error			
Epoch length	-0.0046	0.00056	-8.14	**$2.7x10^{-14}$**	1.20
JME/LEAP	0.017	0.0049	3.57	**0.0004**	1.17
JME/Chowdhury	0.0098	0.0061	1.61	0.12	1.41
Age	-0.00033	0.00026	-1.28	0.20	1.31

N =228, adjusted r^2 =0.25

Supplementary Table 6 - Multiple linear regression model of Brain Network Ictogenicity (BNI) area under the curve (AUC). Control/JME coded as 0/1. Variance inflation factor (VIF) is also reported for each variable.

BNI AUC

Variables	Unstandardized coefficient.		T	P value	VIF
	B	St Error			
Control/JME	2.96	0.57	5.17	**$5.1x10^{-7}$**	1.17
Age	0.11	0.03	3.60	**0.0004**	1.07
Mean epoch length	0.16	0.07	2.11	**0.036**	1.21

N =235, adjusted r^2 =0.13

Biomarker cut off point – low-alpha PSD

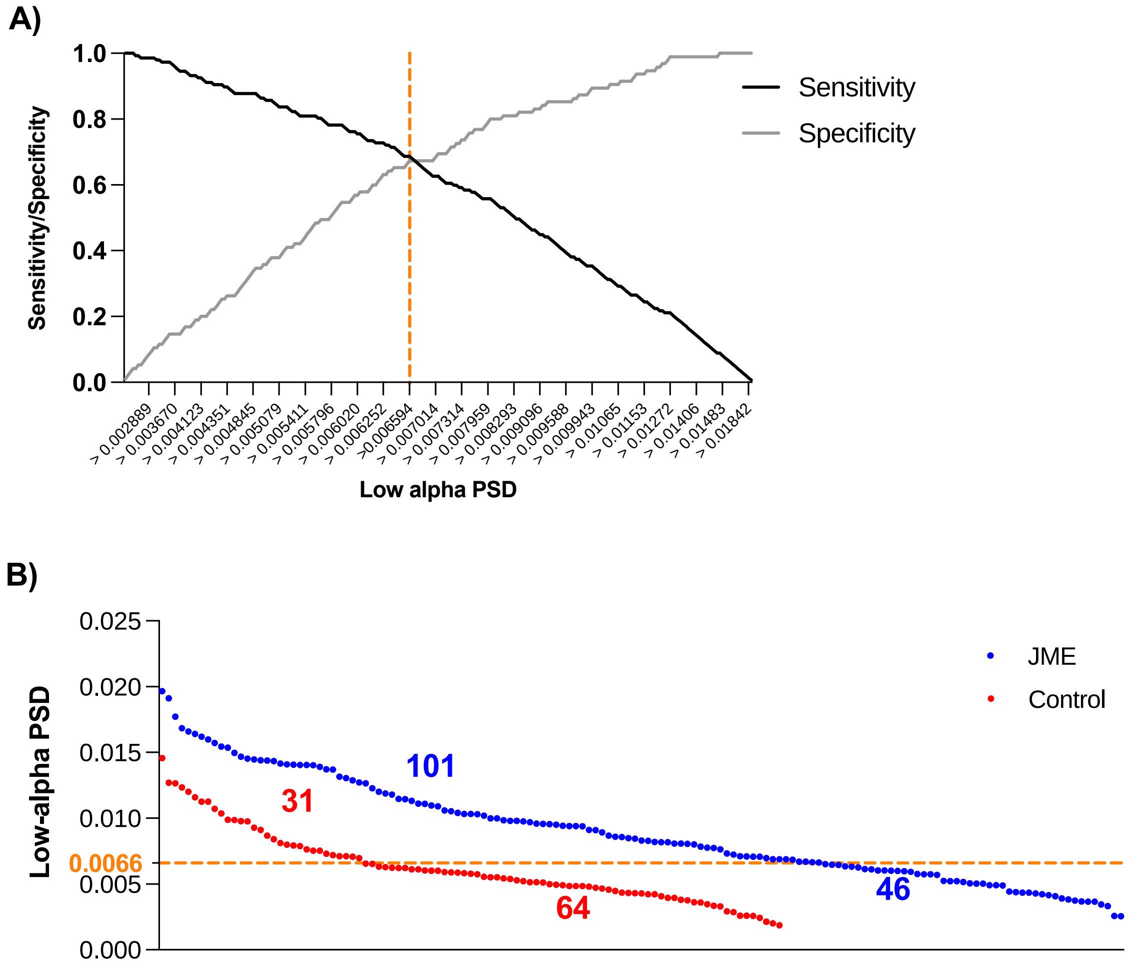

Supplementary Figure 4 - A) A graphical representation of our cut-off choice for pathogenic low-alpha power spectral density (PSD). We chose the cut-off value with the highest sensitivity (0.69) and specificity (0.67). This value is marked by the orange line at 0.0066. **B)** The distribution of low-alpha PSD in JME (blue) and controls (red) and the numbers within the two groups above and below the proposed threshold. Each circle represents an individual. Based on these numbers the Positive Predictive Value=77% and the Negative Predictive Value=58%.

Supplementary Table 7 - Test statistics, exact p-values and N for each correlation displayed in Figure 4. r_s = Spearman's rank coefficient.

EEG features	Age						EEG time						Epoch length		
	JME			Controls			JME			Controls					
	r_s	P-value (2-tailed)	N	r_s	P-value (2-tailed)	N	r_s	P-value (2-tailed)	N	r_s	P-value (2-tailed)	N	r_s	P-value (2-tailed)	N
Delta PSD	-0.53	1.1E-11	141	-0.27	0.01	95	0.01	0.94	138	0.03	0.84	40	-0.13	0.05	242
Low-alpha PSD	0.03	0.74	141	-0.29	0.01	95	-0.19	0.03	138	0.23	0.16	40	-0.14	0.04	242
High-alpha PSD	0.15	0.07	141	0.05	0.60	95	0.22	0.01	138	-0.16	0.33	40	0.16	0.02	242
Beta PSD	0.21	0.01	141	0.45	4.0E-06	95	0.03	0.77	138	-0.11	0.51	40	0.09	0.19	242
Alpha shift	-0.07	0.43	141	-0.15	0.14	95	-0.19	0.03	138	0.28	0.08	40	-0.15	0.02	242
PAF	-0.03	0.70	140	0.25	0.02	93	0.18	0.04	137	-0.38	0.02	40	0.17	0.01	239
Mean strength	0.39	2.0E-06	140	0.24	0.02	95	-0.10	0.25	138	0.06	0.73	40	0.13	0.05	241
Mean strength variance	0.17	0.05	138	-0.18	0.09	94	-0.14	0.10	135	0.16	0.33	40	-0.09	0.19	238
Clustering coefficient	-0.10	0.23	139	-0.36	3.2E-04	94	-0.17	0.06	136	0.14	0.40	40	-0.49	1.1E-15	239
Path length	0.16	0.06	139	-0.15	0.16	92	-0.09	0.29	136	-0.09	0.58	38	-0.02	0.73	237
SMI	-0.28	1.2E-03	137	-0.22	0.04	91	-0.02	0.83	134	0.20	0.24	38	-0.42	2.7E-11	234
BNI	0.37	9.0E-06	140	0.20	0.06	95	-0.13	0.12	137	0.04	0.82	40	0.07	0.28	241

BIOJUME site variation in clustering coefficient

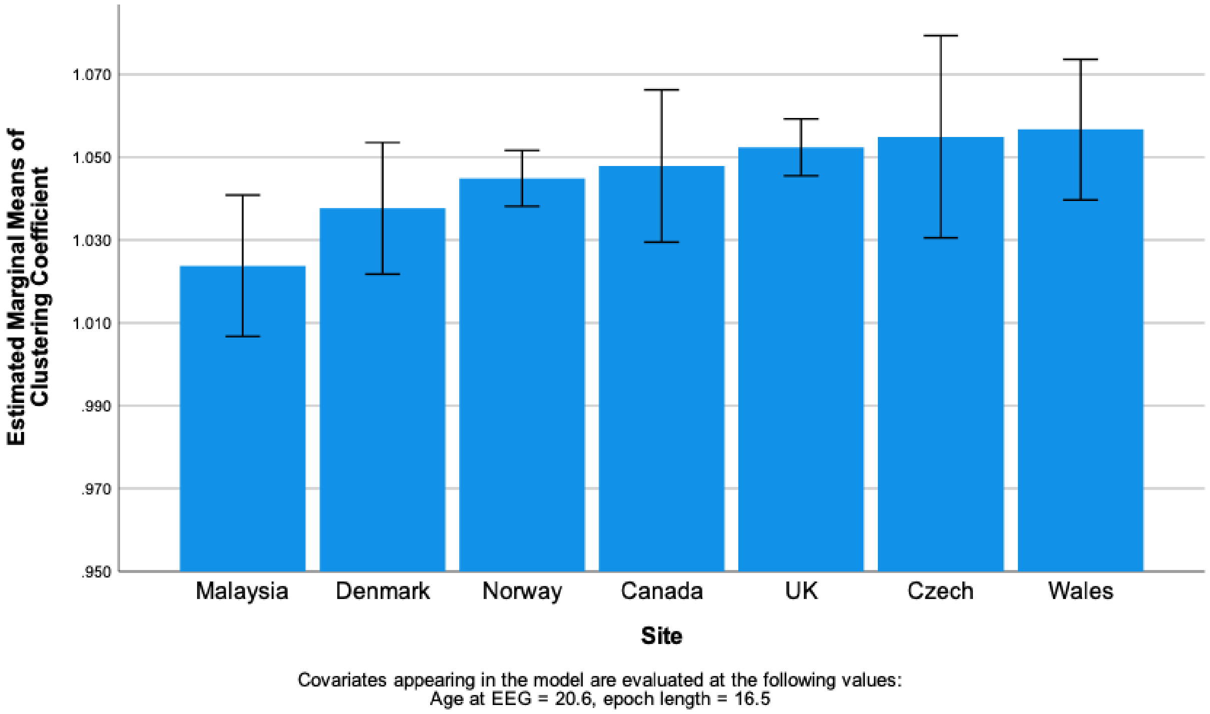

Supplementary Figure 5 - Estimated marginal means of clustering coefficient in JME EEGs stratified by site, controlling for age at EEG and epoch length. There was indication of a difference in clustering coefficient based on EEG recording site [F(6,130)=2.14, p=0.053]. Error bars show 95% confidence intervals.

Supplementary Table 8 - Summary of results from multiple linear regression models of each EEG feature testing for association with seizure phenotypes in JME cohort whilst controlling for age and epoch length. N varied between 117 - 139 depending on available phenotype data for each analysis.

	**Worse seizure control**				
	Unstandardized β coeff	95% CI		Standardized β coeff	P-value
Delta PSD	3E-05	-4E-04	5E-04	0.01	0.90
Low-alpha PSD	-3E-04	-1E-03	5E-04	-0.07	0.46
High-alpha PSD	1E-04	-1E-03	1E-03	0.02	0.87
Beta PSD	1E-04	-5E-05	3E-04	0.12	0.16
Alpha shift	-0.02	-0.11	0.07	-0.04	0.68
Peak alpha frequency	0.02	-0.17	0.22	0.02	0.81
Mean strength	0.04	-0.22	0.31	0.03	0.74
Mean strength variance	-0.26	-0.55	0.03	-0.16	0.07
Clustering coefficient	-0.01	-0.01	-2E-03	-0.20	**0.01**
Path length	-0.01	-0.02	-4E-03	-0.28	**0.002**
Small-world index	2E-03	-0.01	0.01	0.05	0.56
BNI	0.07	-0.77	0.90	0.01	0.88

	**Absence seizure**				
	Unstandardized β coeff	95% CI		Standardized β coeff	P-value
Delta PSD	7E-04	-2E-05	1E-03	0.14	0.06
Low-alpha PSD	-2E-04	-2E-03	1E-03	-0.03	0.77
High-alpha PSD	-9E-04	-3E-03	1E-03	-0.07	0.40
Beta PSD	1E-04	-2E-04	4E-04	0.06	0.51
Alpha shift	0.02	-0.13	0.16	0.02	0.83
Peak alpha frequency	0.07	-0.23	0.39	0.04	0.66
Mean strength	-0.27	-0.70	0.17	-0.10	0.22
Mean strength variance	-0.05	-0.53	0.43	-0.02	0.84
Clustering coefficient	0.00	-0.01	0.01	0.06	0.39
Path length	-0.01	-0.02	2E-03	-0.13	0.13
Small-world index	0.01	5E-04	0.02	0.15	**0.04**
BNI	-0.74	-2.10	0.62	-0.09	0.29

	**Lack of PPR**				
	Unstandardized β coeff	95% CI		Standardized β coeff	P-value
Delta PSD	-9E-05	-1E-03	1E-03	-0.02	0.82
Low-alpha PSD	2E-04	-1E-03	2E-03	0.03	0.79
High-alpha PSD	-2E-04	-2E-03	2E-03	-0.02	0.87
Beta PSD	-7E-05	-4E-04	3E-04	-0.04	0.68

Alpha shift	0.01	-0.15	0.17	0.01	0.93
Peak alpha frequency	0.05	-0.30	0.40	0.03	0.77
Mean strength	0.20	-0.28	0.68	0.07	0.41
Mean strength variance	-0.22	-0.67	0.24	-0.09	0.35
Clustering coefficient	-0.01	-0.02	-3E-04	-0.15	**0.04**
Path length	-4E-03	-0.01	0.01	-0.07	0.45
Small-world index	-0.01	-0.02	0.01	-0.08	0.32
BNI	0.24	-1.23	1.72	0.03	0.75

No morning predominance of seizures

	Unstandardized β coeff	95% CI		Standardized β coeff	P-value
Delta PSD	9E-04	5E-05	2E-03	0.15	**0.04**
Low-alpha PSD	3E-04	-1E-03	2E-03	0.03	0.73
High-alpha PSD	-2E-03	-4E-03	1E-03	-0.10	0.24
Beta PSD	-8E-05	-4E-04	3E-04	-0.04	0.63
Alpha shift	0.07	-0.10	0.25	0.07	0.41
Peak alpha frequency	-0.01	-0.38	0.35	-0.01	0.94
Mean strength	-0.28	-0.80	0.25	-0.09	0.30
Mean strength variance	-0.30	-0.86	0.27	-0.09	0.30
Clustering coefficient	0.01	-3E-03	0.02	0.10	0.17
Path length	-0.01	-0.02	4E-03	-0.12	0.18
Small-world index	0.02	0.01	0.03	0.20	**0.01**
BNI	-0.68	-2.31	0.94	-0.07	0.41

Triggered seizures

	Unstandardized β coeff	95% CI		Standardized β coeff	P-value
Delta PSD	-5E-04	-1E-03	2E-04	-0.10	0.18
Low-alpha PSD	1E-04	-1E-03	2E-03	0.01	0.89
High-alpha PSD	2E-05	-2E-03	2E-03	0.00	0.98
Beta PSD	1E-04	-2E-04	4E-04	0.07	0.43
Alpha shift	-0.01	-0.18	0.16	-0.01	0.89
Peak alpha frequency	0.04	-0.32	0.39	0.02	0.84
Mean strength	0.00	-0.49	0.49	0.00	0.99
Mean strength variance	-0.33	-0.86	0.20	-0.11	0.22
Clustering coefficient	-0.01	-0.14	0.01	-0.07	0.32
Path length	-0.01	-0.03	2E-03	-0.21	**0.02**
Small-world index	0.01	0.00	0.02	0.12	0.11
BNI	-0.01	-1.53	1.51	0.00	0.99

Association of seizure prognosis with EEG features - alternative definition

Sodium valproate is the most effective ASM in JME,[19] however many females are not prescribed this ASM due to its teratogenic effect. To investigate whether a lack of sodium valproate prescription was influencing the results of association of EEG features with seizure prognosis, we further stratified the JME cohort testing for associations of seizure prognosis with EEG features in only those who had used sodium valproate. This encompassed 60% of all cases, 80% of males (n=43) and 48% of females (n=39).

The results from linear regression models in this smaller subset of participants are presented in Supplementary Table 9. We see that the association of poorer seizure control with decreased path length remains (β=-0.008 (standardised β = -0.24), p=0.035), as in the original sample (Supplementary Table 8). The association with decreased clustering coefficient decreases compared to the original sample (β=-0.004 (standardised β = -0.10), p=0.33). Unlike in the original sample, increased relative beta PSD is associated with worse seizure control in this smaller subset (β=0.0002 (standardised β = 0.23), p=0.049), however only with a very small effect. Results remain similar to the original sample for all other EEG features.

Supplementary Table 9 -Table standardized beta coefficients and p-values of linear regression models of associations seizure prognosis with EEG features in the subset of JME cohort (n=83) who have used sodium valproate to control their seizures, controlling for age and epoch length. BNI = Brain Network Ictogenicity; PSD = Power spectral density.

EEG feature	**Association with (worse) seizure control**				
	Unstandardized β coeff.	95% CIs		Standardized β coeff.	P-value
Delta PSD	-7.6E-5	-0.001	0.001	-0.03	0.80
Low-alpha PSD	-0.001	-0.002	0.001	-0.12	0.32
High-alpha PSD	0.001	-0.001	0.002	0.06	0.59
Beta PSD	0.0002	1.0E-7	0.0004	0.23	**0.05**
Alpha shift	-0.055	-0.18	0.07	-0.10	0.39
Peak alpha frequency	0.168	-0.08	0.42	0.15	0.19
Mean strength	0.053	-0.28	0.39	0.04	0.76
Mean strength variance	-0.29	-0.65	0.06	-0.19	0.11
Clustering coefficient	-0.004	-0.01	0.004	-0.10	0.33
Path length	-0.008	-0.02	-0.001	-0.24	**0.04**
Small-world index	0.004	-0.004	0.01	0.09	0.36
BNI	0.26	-0.78	1.31	0.06	0.62
